# Loop region mutations in Omp33-36: unveiling reduced antimicrobial permeability and resistance in *Acinetobacter baumannii* through structural modeling and membrane-protein simulations

**DOI:** 10.3389/fmicb.2026.1954565

**Published:** 2026-10-20

**Authors:** Subhasree Roy, Khalid Ansari, Mousumi Hazra, Vivek Junghare, Tanusree Das, Arup Samanta, Sulagna Basu, Saugata Hazra

**Affiliations:** 1 Division of Bacteriology, ICMR National Institute for Research in Bacterial Infections (ICMR-NIRBI), Kolkata, India; 2 Department of Bioscience and Bioengineering, Indian Institute of Technology Roorkee (IITR), Roorkee, India; 3 Department of Physics, Indian Institute of Technology Roorkee (IITR), Roorkee, India; 4 Department of Botany and Microbiology, Gurukula Kangri (Deemed to Be University), Haridwar, India; 5 Centre of Excellence for Chemical Mechanisms of Life, Uppsala University, Uppsala, Sweden; 6 Centre for Nanotechnology, Indian Institute of Technology Roorkee (IITR), Roorkee, India

**Keywords:** *Acinetobacter baumannii*, antimicrobial resistance, loop mutations, membrane-protein simulation, Omp33-36, reduced permeability, structure modeling

## Abstract

**Background:**

*Acinetobacter baumannii* can reduce its susceptibility to antimicrobials by altering the permeability of the outer membrane, e.g., by changing the abundance and function of porins. Omp33-36 has been implicated in susceptibility to multiple antibiotics including carbapenems, but the structural effect of sequence variations observed in clinical isolates is poorly understood. We investigated whether substitutions in Omp33-36 from multidrug-resistant clinical isolates affect porin dynamics and contribute to permeability-related resistance.

**Methods:**

Multilocus sequence typing identified lineages and Sanger sequencing identified Omp33-36 substitutions in *A. baumannii* bloodstream isolates. The expression of *omp33-36* was measured by reverse transcription quantitative polymerase chain reaction (RT-qPCR) and the acquired resistance determinants were analyzed by PCR. Based on the experimentally determined structure (PDB 6GIE), we built full-length wild-type and variant models of Omp33-36, embedded them into a phospholipid bilayer and performed all-atom molecular dynamics simulations to study their conformational behavior, residue mobility and loop-associated interactions.

**Results:**

Two multidrug-resistant isolates with reduced *omp33-36* expression harbored substitutions Phe125Asn/Glu243Ser (A_146) and Gly248Asp/Asp250Asn (A_106). The β-barrel scaffold was stable, but substitutions affected local packing, interaction network and loop dynamics. Phe125Asn reduced steric constraints and allowed more polar interactions. Mutations in the Gly242-Gly252 region created new local and cross-region contacts that constrained conformational freedom. These changes favored conformational states with increased mechanical constraints and without global destabilization. The observations are consistent with a working two-gate coupled motion model in which extracellular- and periplasmic-facing access regions are dynamically coupled components. The mutation-associated change in loop dynamics and preference for less-accessible channel states may disrupt coordination, but no direct evidence for diminished antibiotic flux was provided.

**Conclusion:**

Substitutions in Omp33-36 alter local networks of interactions and loop dynamics but preserve the β-barrel architecture. These changes and a reduced expression of porins may result in changes in permeability. However, the presence of other resistance determinants makes Omp33-36 changes a possible contributor to a multifactorial resistance phenotype rather than an independently demonstrated cause. Direct measurements of permeability and isogenic studies with expression control are necessary to validate the proposed mechanism.

## 1 Introduction

*Acinetobacter baumannii* has emerged as a major global public health threat because of its increasing antimicrobial resistance and its association with severe infections in critically ill patients. Its resistance is mediated by multiple mechanisms, including antibiotic-inactivating enzymes, reduced outer membrane permeability, active efflux, and alterations in antimicrobial targets. Carbapenem resistance is primarily driven by acquired OXA-type carbapenemases, as well as by metallo-β-lactamases. The interplay of these mechanisms is responsible for the multidrug-resistant phenotype observed in *A. baumannii* (Dubey et al., 2025). In view of this pathogen’s public health threat because of the high antibiotic resistance in *A. baumannii*, World Health Organization has defined carbapenem-resistant *A. baumannii* as a critical priority pathogen (World Health Organization, 2024).

Among the non-enzymatic mechanisms, outer membrane proteins (OMPs), including porins, regulate the permeability of the outer membrane of Gram-negative bacteria and facilitate the uptake of small molecules, including nutrients and antimicrobial agents (Nikaido and Vaara, 1985; Nikaido, 2003; Fernández and Hancock, 2012). Porins, which are proteins that make channels, were first found in *Escherichia coli* in 1976 (Nakae, 1976). Since then, porins have been extensively studied in pathogens like *Escherichia coli*, *Klebsiella pneumoniae*, *A. baumannii*, *Pseudomonas aeruginosa Neisseria gonorrhoeae, Serratia marcescens, Proteus vulgaris, Salmonella* spp etc., (Nikaido and Vaara, 1985; Fernández and Hancock, 2012; Zhou et al., 2023). Porins can be categorized into two groups: (i) nonspecific general diffusion channels, which let small solutes pass through without selecting a substrate, and (ii) substrate-specific porins, which have low-affinity binding sites for certain compounds (Nikaido and Vaara, 1985; Fernández and Hancock, 2012; Zhou et al., 2023).

Porins are β-barrel proteins made up of anti-parallel β-strands, with loops on the outside and α-helices in the middle. Their hydrophilic pores and constriction zones control solute diffusion, pore size, and ion selectivity, ensuring permeability while the membrane remains intact. The extracellular loops that connect β-strands at the membrane surface are very important for shaping pore geometry and controlling selectivity. Porins usually come together as homotrimers, with each monomer making its own channel. The pore lumen is lined with amino acids that attract water, which helps polar molecules move through it. Hydrophobic or aromatic residues (like phenylalanine and leucine) hold the porin in place within the lipid bilayer (Mayse and Movileanu, 2023). The constriction zone, which is in the middle of the pore, has polar and charged residues that control the flow of ions and the choice of substrates. Hydrophilic residues facilitate the movement of polar solutes through a solution. Charged residues like lysine, arginine, aspartate, and glutamate control how ions move through a solution (Vinothkumar and Henderson, 2010). Porins usually let molecules up to about 600 Da through, with a preference for hydrophilic ones. OmpF, for instance, lets molecules diffuse in general, while LamB (maltoporin) is good at taking in maltose. Hydrophilic and charged residues in porins have a big impact on how well antibiotics can get through (Prajapati et al., 2021).

Changes in porin expression or mutations can significantly affect how resistant Gram-negative bacteria are to antibiotics. Porins directly limit the flow of antibiotics by reducing pore size, altering the electrostatic potential, or lowering expression (Phan and Ferenci, 2017; Breijyeh et al., 2020; Zhou et al., 2023). There are many well-known examples that show this principle. In Enterobacterales, the downregulation of OmpF and/or OmpC diminishes β-lactam permeability (Lavigne et al., 2012; Majewski et al., 2016; Babouee Flury et al., 2016; Zhou et al., 2023). In *E. coli* and *K. pneumoniae*, the loss or mutation of OmpF and OmpC impedes cephalosporin entry, frequently leading to clinical treatment failures (Lou et al., 2011; Liu et al., 2012; Ziervogel and Roux, 2013; Choi and Lee, 2019; Hakkinen et al., 2020). Under stress, OmpF suppression makes quinolones less effective, while the loss of OmpK35/OmpK36 in *K. pneumoniae* is strongly linked to carbapenem resistance (Hamzaoui et al., 2018). OmpW downregulation also makes *E. coli* resistant to kanamycin and *Salmonella* resistant to ceftriaxone (Hong et al., 2006; Li et al., 2015). *E. coli* that doesn’t have OmpX is resistant to hydrophobic antibiotics like amikacin, gentamicin, novobiocin, and nalidixic acid (Otto and Hermansson, 2004). In *Pseudomonas aeruginosa*, porin OprD facilitates the uptake of β-lactams. Mutations that modify pore size or charge impede carbapenem entry and induce high-level resistance (Prajapati et al., 2021). In a similar way, the loss of OmpK35/36 in *K. pneumoniae* makes the membrane less permeable to different antibiotics by working with other mechanisms (Brunson et al., 2019; Sugawara et al., 2016). In *N. gonorrhoeae*, PorB mutations correlate to decreased susceptibility to penicillins and cephalosporins (Osawa et al., 2017; Thakur et al., 2018). These studies collectively showed that the loss or mutation of porins not only diminishes antibiotic penetration but also often engages efflux systems and periplasmic β-lactamases, resulting in a multifaceted resistance phenotype.

In *A. baumannii*, porins also contribute to multidrug resistance. Changes in porins lower permeability and make treatment less effective (Galdiero et al., 2012; Novović et al., 2018). The OmpA is one of the best-studied porins. It is involved in the development of antimicrobial resistance in *A. baumannii* mutant strains lacking OmpA (Sugawara and Nikaido, 2012; Iyer et al., 2018; Uppalapati et al., 2020; Kyriakidis et al., 2021; Castanheira et al., 2023). CarO, AbuO, OprD, and Omp33-36 are the other examples of porins associated with antimicrobial resistance in *A. baumannii* (Morán-Barrio et al., 2017; Novović et al., 2018; Uppalapati et al., 2020; Castanheira et al., 2023). CarO mutations and decreased expression are directly associated with carbapenem resistance (Limansky et al., 2002; Mussi et al., 2005, 2007; Catel-Ferreira et al., 2011; Dafopoulou et al., 2015; Zhu et al., 2019). Role of AbuO in conferring broad-spectrum antimicrobial resistance in *A. baumannii* has also been reported earlier (Srinivasan et al., 2015), whereas OprD was first identified during outer membrane investigations of carbapenem-resistant *A. baumannii* (Uppalapati et al., 2020).

Omp33-36 is associated with drug resistance including carbapenem resistance in *A. baumannii* by reducing antibiotic permeability (Clark, 1996; del Mar Tomás et al., 2005; Novović et al., 2018). Moreover, insertion sequence-mediated disruption and inactivation of *omp33-36* have been repeatedly reported in clinical isolates of *A. baumannii* and may contribute to the development of resistance phenotype (Smani et al., 2013; Dubey et al., 2025). Crystal structure analysis showed that this protein comprises a 14-β-stranded barrel, six periplasmic turns and seven extracellular loop which do not form stable constrictions (Abellón-Ruiz et al., 2018; Jahangiri et al., 2018). On the other hand, an extended periplasmic turn connecting β-strands 2 and 3 folds into the pore lumen and completely blocks the aqueous channel. Their study showed how *A. baumannii* outer membrane proteins contribute to the low permeability of the cell envelope of this bacterium. However, efforts toward structural characterization of the porin Omp33-36 have still lagged because of the hydrophobic nature and low solubility of membrane proteins as compared to soluble cytosolic proteins. Consequently, the correlation between its structural features and functional properties remains incompletely understood.

Porins represent a delicate balance between permeability, selectivity, and stability. Porin mutations are well known to confer resistance, but changes in Omp33-36, especially in the extracellular loop regions that define pore geometry, have not been systematically documented. Changes in these loops could narrow the channel, alter its electrostatic profile, or affect its dynamic flexibility. This would make it harder for antibiotics to get in and make the bacteria resistant to more than one drug. However, detailed mechanistic insights are still lacking, leaving the precise role of Omp33-36 in antibiotic resistance poorly understood. The present study focuses on mutations in the Omp33-36 loop region in two clinical strains of *A. baumannii* isolated from the blood of septicemic neonates. To explore how such mutations might influence pore architecture and function, comparative three-dimensional modeling and molecular dynamics simulations were employed. These computational approaches were used to predict possible structural alterations, assess changes in pore dynamics within a membrane-like environment, and provide insights into the relationship between Omp33-36 structure and antibiotic permeability. To our knowledge, this is the first attempt to model the full-length Omp33-36 embedded in a lipid bilayer, aiming to advance understanding of its potential role in multidrug resistance and its value as a therapeutic target.

## 2 Materials and methods

### 2.1 Bacterial isolates, antibiotic susceptibility and multi locus sequence typing

For this study, two multidrug-resistant (MDR) *A. baumannii* isolated from the blood of septicemic neonates admitted to a neonatal intensive care unit of a tertiary care center of India were selected, which also showed lower expression of *omp33-36* and which correspondingly had amino acid mutations in porin Omp33-36. The identification of isolates as *A. baumannii* was performed using the VITEK 2 compact system (BioMérieux, Marcy l’Etoile, France) and confirmed by assignment of the isolates to reference STs (Sequence Types) included in *A. baumannii* isolates database as described in Migliaccio et al. (2023). MIC (Minimum Inhibitory Concentration) of different antimicrobials was determined by VITEK 2 compact system (bioMérieux, Marcy-l’Étoile, France) and interpreted according to CLSI (Clinical and Laboratory Standards Institute [CLSI], 2025) breakpoints. Sequence Types (STs) of the isolates were determined using the Pasteur Multilocus Sequence Typing (MLST) Scheme^1^.

### 2.2 Identification of antimicrobial resistance determinants

PCR was carried out for carbapenem resistance genes (*bla*_IMP, VIM, SPM, GIM, SIM, NDM_ and *bla*_OXA-23-like, OXA-24-like, OXA-58-like_), extended spectrum-β-lactamase genes (*bla*_CTX–M, VEB, PER_), *bla*_AmpC_ genes, aminoglycoside resistance genes (*rmtA, rmtB, rmtC, rmtD, armA*) and fluoroquinolone resistance genes (*qnrA, qnrB, qnrS*, and *aac(6′)-Ib-cr*) (Chatterjee et al., 2016; Roy et al., 2021).

### 2.3 Reverse transcription quantitative PCR (RT-qPCR)

Total RNA was extracted from the bacterial cultures using a Nucleospin RNA isolation kit (Nucleospin, Macherey-Nagel, DÜren). Reverse transcription was performed with 2 μg of RNA according to the manufacturer’s instructions using the high-capacity cDNA reverse transcription kit (Applied Biosystems, Warrington, UK). The target gene expression was quantified with Power SYBR Green PCR Master Mix (Applied Biosystems) using the StepOne Plus Real-Time PCR System and software (Applied Biosystems, USA) according to the manufacturer’s instructions. Primers (F: 5′CAAGTGTTGCTAACCAATTCGCT3′ and R: 5′ GTTTTCTTGACCGAATGCACC3′) were used as described previously (Rumbo et al., 2013). The relative gene expression level (△CT) of *omp33-36* gene was calculated against that for the 16S rRNA gene and the △△CT was calculated against that for the susceptible strain *A. baumannii* ATCC 19606 which served as the control (expression = 1). The relative expression level of the *omp33-36* was subsequently calculated using the 2^–△△CT^ method.

### 2.4 Identification of amino-acid substitutions in Omp33-36

Sanger sequencing of *omp33-36* was carried out using the primers (F: 5′ ATGAAAAAATTGGTTTAGCCAC3′ and R: 5′AGAAACGGAATTTAGCA3′) and condition published previously (Novovic et al., 2015). The protein sequences were compared with reference strain *A. baumannii* ATCC 19606 to identify the mutations within Omp33-36.

### 2.5 Structural modeling of Omp33-36

The Omp33-36 protein sequence of the reference strain *A. baumannii* ATCC 19606 (WP_000733005.1) was used for identification of an appropriate structural template. A search against experimentally determined structures in the Protein Data Bank identified PDB ID 6GIE as a suitable structural reference (Abellón-Ruiz et al., 2018). PDB 6GIE corresponds to the experimentally determined *A. baumannii* outer membrane protein Omp33 (Omp33/33-36), solved by X-ray crystallography at 2.10 Å resolution. The deposited protein sequence comprises 296 residues, of which 218 residues are experimentally solved in the crystal structure. The structural correspondence of 6GIE to the Omp33-36 target, together with its experimental determination from *A. baumannii*, supported its selection as the template for comparative modeling. The target sequence was subsequently used to generate a full-length Omp33-36 structural model using SWISS-MODEL server (Waterhouse et al., 2018). Regions not represented in the experimentally resolved portion of the template were modeled as part of the comparative-modeling procedure. The mutations identified in the clinical isolates (by sanger sequencing) were subsequently introduced into the reference model using the Simple Mutation tool in COOT (Emsley and Cowtan, 2004). Structural visualization and analysis were performed using PyMOL and COOT, and the resulting models were evaluated using PROCHECK (Laskowski et al., 2018).

### 2.6 System preparation steps for DPPC membrane simulation of Omp33-36

Following protein structure modeling, the lipid bilayer was modeled, opting for the DPPC (dipalmitoylphosphatidylcholine) variant for simulation. Initially, a DPPC lipid bilayer comprising 128 lipids was employed, with subsequent protein insertion into the bilayer. Before orienting the protein in the lipid bilayer, the lipid bilayer was first energy-minimized to remove steric clashes and relax any unfavorable lipid conformations, providing a stable membrane environment for protein insertion and orientation. Subsequently, water and ions were added to solvate and neutralize the system, utilizing the TIP3P (transferable intermolecular potential) water model and Na^+^ and Cl^–^ ions to mimic physiological conditions, as enumerated in Supplementary Table 1. After structural modeling, the choice of force field for proteins was guided by the selection of GROMOS96/53a6 (GROningen MOlecular Simulation) force field. Additionally, the GROMOS96/53a6 force field was adapted for lipid by integrating lipid parameters (atom types, nonbonded, pair types, and dihedral types), thus serving as the force field for lipids in the simulations (Tieleman and Berendsen, 1998; Varma et al., 2006; Bond and Sansom, 2003; Sousa et al., 2022).

### 2.7 Protein orientation in the membrane

The Omp33-36 structure was oriented and inserted into the lipid bilayer based on the experimentally determined topology and membrane orientation of the experimentally solved Omp33-36 structure used as the structural reference. The orientation was chosen so that the β-barrel would be inserted in the right way and the extracellular and periplasmic loop regions would be correctly oriented prior to the membrane construction and the subsequent molecular dynamics simulations.

### 2.8 Energy minimization steps for molecular dynamics simulation

Following system preparation, it is imperative to conduct system minimization to mitigate any potential steric clashes between protein and lipid atoms. The steepest descent algorithm for minimization was used to monitor the minimization steps, verifying convergence by plotting the energy graph using Grace Software (Bond and Sansom, 2003).

### 2.9 Equilibration steps for molecular dynamics simulation

After energy minimization of the system, the two stages of equilibration were run to confirm that the system must get stabilized. Two types of equilibrations were used (1) NVT equilibration (constant-temperature, constant-volume ensemble) which indicates the number of particles (N), volume (V), and temperature (T) must be constant. (2) NPT equilibration (constant-temperature, constant-pressure ensemble) which indicates the number of particles (N), pressure (P), and temperature (T) must be constant. A temperature of 323 K was used for the molecular dynamics simulations to facilitate conformational sampling within the available simulation timescale. This temperature is higher than the physiological temperature relevant to bacterial growth and therefore does not directly represent the native physiological environment of *A. baumannii*. The wild-type and mutant Omp33-36 systems were simulated under identical temperature and simulation conditions, the comparative differences in RMSD (Root Mean Square Deviation), RMSF (Root Mean Square Fluctuation), radius of gyration, and membrane-associated behavior remain informative for assessing the relative structural and dynamic effects of the mutations. The use of 323 K is therefore considered a methodological choice for enhanced sampling rather than a physiological representation of the protein environment. At first, NVT equilibration was performed by heating the system from 0 to 323 K using leap-frog integrator for the 100 picoseconds duration with step size of 2 femtoseconds using constraint algorithm LINCS (LINear Constraint Solver) which is a holonomic constraint. For NVT equilibration, a V-rescale thermostat (modified version of Berendsen thermostat) was used for temperature coupling. Prior to proceeding with the subsequent equilibration step, we ensured the stabilization of the system’s temperature at 323 K by plotting the data. NPT equilibration was performed at 1 bar using leap-frog integrator for the 1 nanosecond (1000 picoseconds) duration with step size of 2 femtoseconds using constraint algorithm LINCS (Hess et al., 1997). For NPT equilibration, Nose-hoover thermostat was used for temperature coupling and Parrinello-Rahman barostat was used for the pressure coupling (Nosé, 1984; Hoover, 1985; Parrinello and Rahman, 1981; Nosé and Klein, 1983). Both equilibrations were performed under long-range electrostatic conditions using coulomb type as Particle Mesh Ewald (PME). Van der Waals cut-off (r_*vdw*_) and electrostatic cut-off (r_*coulomb*_) were used at 1.2 nm for the short-range. We also plotted the systems pressure, which was stabilized at 1 bar.

### 2.10 Molecular dynamics production run

The final step entailed executing the “MD run (Molecular Dynamics run)”, commonly referred to as the production run. Following all minimization and equilibration procedures, MD run was initiated utilizing a leap-frog integrator, spanning a duration of 100 nanoseconds with a step size of two femtoseconds. Throughout this simulation, coordinate and velocity data were saved at intervals of every two picoseconds. Additionally, XTC compressed (GROMACS compressed) trajectory files and energy were saved after every two picoseconds. The preservation of XTC trajectory and coordinates enables the examination of the dynamic behavior of the simulated model. Bond constraints were imposed, utilizing LINCS for holonomic constraints, while all other parameters remained consistent with those specified for NPT equilibration. The recorded trajectories serve as the basis for subsequent analysis. Upon completion of the simulation, trajectories were visualized using PyMOL and VMD (Visual Molecular Dynamics) tools (Humphrey et al., 1996), facilitating thorough analysis. However, some of the 128 lipids which were initially selected for system preparation overlapped with the protein coordinates, leading to hindrances in energy minimization and rendering the system unstable. To rectify this and maintain the area per lipid within mandatory limits, we imposed restrictions and removed select lipids obstructing protein-lipid interaction. The resulting number of lipids utilized for the MD run post-deletion is outlined in Supplementary Table 1. Initially, solvent molecules were introduced into the system using TIP3P, occupying all unoccupied areas within the simulation box. A Perl script was utilized to selectively delete water molecules in proximity to the protein and between bilayers to ensure the absence of water molecules between proteins and bilayers. Consequently, as detailed in Supplementary Table 1, a specific number of water molecules remained above and below the lipid bilayer leaflets post-deletion.

### 2.11 Structural modeling and conservation analysis of Omp33-36 and porin-like proteins

In this study, we have examined a plethora of porins from several Gram-negative organisms and represented 23 optimized ones with their structures to emphasize the shared architectural characteristics. The 23 structures were modeled as part of this study to obtain full-length protein structures. If experimental structures were available, we used the corresponding PDB structures as templates. Since some of the PDB structures were incomplete or covered only part of the protein, the missing regions were modeled to generate full-length structures. For proteins without suitable experimental structures, AlphaFold models were used as the starting structures. SWISS-MODEL was used to generate the structural models based on suitable template structures. These full-length structures were then optimized and used for the subsequent analysis.

### 2.12 Comparative sequence and evolutionary conservation analysis of Omp33-36 among *Acinetobacter* species

Evolutionary conservation of Omp33-36 residues among the *Acinetobacter* spp. was assessed using the ConSurf server, and conservation scores were mapped onto the Omp33-36 amino-acid sequence and three-dimensional structure (Ashkenazy et al., 2016). Particular attention was given to residues 125, 243, 248, and 250, corresponding to the substitutions identified in the study isolates. ConSurf classification was used to distinguish variable and conserved residues and to identify conserved exposed or buried positions.

For comparison with epidemic *A. baumannii* lineages, Omp33-36 amino-acid sequences representing International Clone 1 (IC1/ST1), International Clone 2 (IC2/ST2), and International Clone 8 (IC8/ST10) were compared with the reference Omp33-36 sequence (WP_000733005.1) and the study isolates. Multiple-sequence alignment was performed using MultAlin, and the alignment was visualized and annotated using ESPript. Residues 125, 243, 248, and 250 were specifically examined to determine their conservation and occurrence across the different clonal backgrounds (Corpet, 1988; Gouet et al., 1999).

## 3 Results

### 3.1 Bacterial isolates, gene expression of *omp33-36*, and analysis of mutations

This study involved two *A. baumannii* bloodstream isolates, A_146 (ST1406) and A_106 (ST1488), both obtained from blood of septicemic neonates. ST1488 allelic profile has been identified already as *A. baumannii* in *Acinetobacter* PubMLST database, and ST1406 is a single locus variant of ST575, which is identified in *Acinetobacter* PubMLST database as *A. baumannii*. The isolates were detected as MDR, exhibiting resistance to three or more antimicrobial classes (Magiorakos et al., 2012). Antimicrobial Susceptibility Testing revealed that both isolates were resistant to several antibiotics, including ceftazidime, cefepime, aztreonam, amikacin, and ciprofloxacin (Table 1). The distinct sequence types of these isolates enabled us to investigate potential variations in the structure and dynamics of Omp33-36 across genetically diverse MDR *A. baumannii* strains belonged to ST1406 and ST1488 (Table 1). Genotypic screening revealed the presence of *bla*_PER_, which imparts resistance to extended-spectrum cephalosporins and *aac(6′)-Ib-cr*, associated with aminoglycoside and fluoroquinolone resistance, in both samples.

**TABLE 1:** Minimum inhibitory concentration (MIC), multi locus sequence typing (MLST), amino acid mutations within Omp33-36 detected by sanger sequencing and expression levels (fold change) of Omp33-36 detected by reverse transcription quantitative PCR (RT-qPCR) of the two multidrug-resistant *Acinetobacter baumannii.*

Strain No	MLST	MIC (mg/L)	Amino acid mutations in Omp33-36	*omp33-36* fold change
A_146	ST1406	CAZ (≥64); FEP (≥64); ATZ (≥64); DOR (0.5); IMP (1); MEP (1); AMK (≥64); CIP (≥4); MINO (2), COL (≤0.5)	F125N, E243S	0.3
A_106	ST1488	CAZ (≥64); FEP (≥64); ATZ (≥64); DOR (≤0.12); IMP (1); MEP (1); AMK (≥64); CIP (≥4); MINO (≤1), COL (≤0.5)	G248D, D250N	0.2

CAZ, ceftazidime; FEP, cefepime; ATZ, aztreonam; DOR, doripenem; IMP, imipenem; MEM, meropenem; AMK, amikacin; CIP, ciprofloxacin; MINO, minocycline; COL, colistin; F, phenylalanine; N, asparagine; E, glutamic acid; S, serine; G, glycine; D, aspartic acid.

The RT-qPCR analysis showed that the expression of *omp33-36* was significantly reduced in both the study isolates compared to *A. baumannii* ATCC 19606 (Table 1). To investigate the potential structural changes in the porin, the *omp33-36* gene was amplified and sequenced by sanger sequencing. Sequence alignment identified several amino acid mutations: isolate A_146 (ST1406) carried Phe125Asn and Glu243Ser substitutions, whereas isolate A_106 (ST1488) exhibited Gly248Asp and Asp250Asn substitutions (Table 1).

### 3.2 Structural modeling of Omp33-36 variants in multidrug-resistant *A. baumannii* for insights into antibiotic permeability

The Ramachandran plot analysis for structural validation indicated that over 90% of the residues in all Omp33-36 models were located in the most favored and allowed regions, thereby confirming the good stereochemical quality of the predicted structures. The validated models were thus considered suitable starting structures for the subsequent molecular dynamics simulations (Supplementary Figure 1).

The porin sequence from the reference strain *A. baumannii* ATCC 19606 was employed as a template for structural modeling of the Omp33-36 variants identified in the MDR study isolates. This comparative modeling approach was used to examine how sequence-level modifications could potentially alter the three-dimensional architecture of the porin and may influence its permeability function.

To explore these potential structural effects, experiments were designed to assess conformational changes, including possible steric occlusion, alterations in hydrogen-bonding patterns, and variations in loop flexibility within the β-barrel scaffold and loop domains. Particular focus was placed on solvent-exposed loops, as modifications in these regions might influence inter-loop interactions or stabilize conformations that could restrict pore flexibility and alter antibiotic entry. Mapping residue substitutions onto the structural framework and examining the channel’s size, shape, and electrostatic characteristics provided a framework for understanding how such changes could affect porin function.

In this study, we examined porins from diverse Gram-negative organisms and analyzed 23 optimized structures to highlight their common architectural features (Figure 1 and Supplementary Table 2). For a broader evolutionary context, the wild-type Omp33-36 model from *A. baumannii* ATCC 19606 was compared with representative porins from other Gram-negative bacteria (Supplementary Figure 2 and Supplementary Table 2). Figure 1 showed the structural context of Omp33-36 and emphasized the importance of its loops in shaping the channel architecture. Omp33-36 has a β-barrel framework, similar to other outer-membrane porins from Gram-negative bacteria such as OmpF and OprD. However, the architecture and organization of the extracellular loops are rather different between these proteins. In classical porins like OmpF, the formation and constriction of the aqueous channel involve loop regions, with loop L3 reaching into the pore to form an important part of the interior of the channel. OprD also has loop regions important for its structure, contributing to the architecture of the substrate-selective channel. In Omp33-36, the loop regions constitute a prominent and relatively complex component of the channel-entry environment, with extended loop features influencing access to the pore.

**FIGURE 1:**
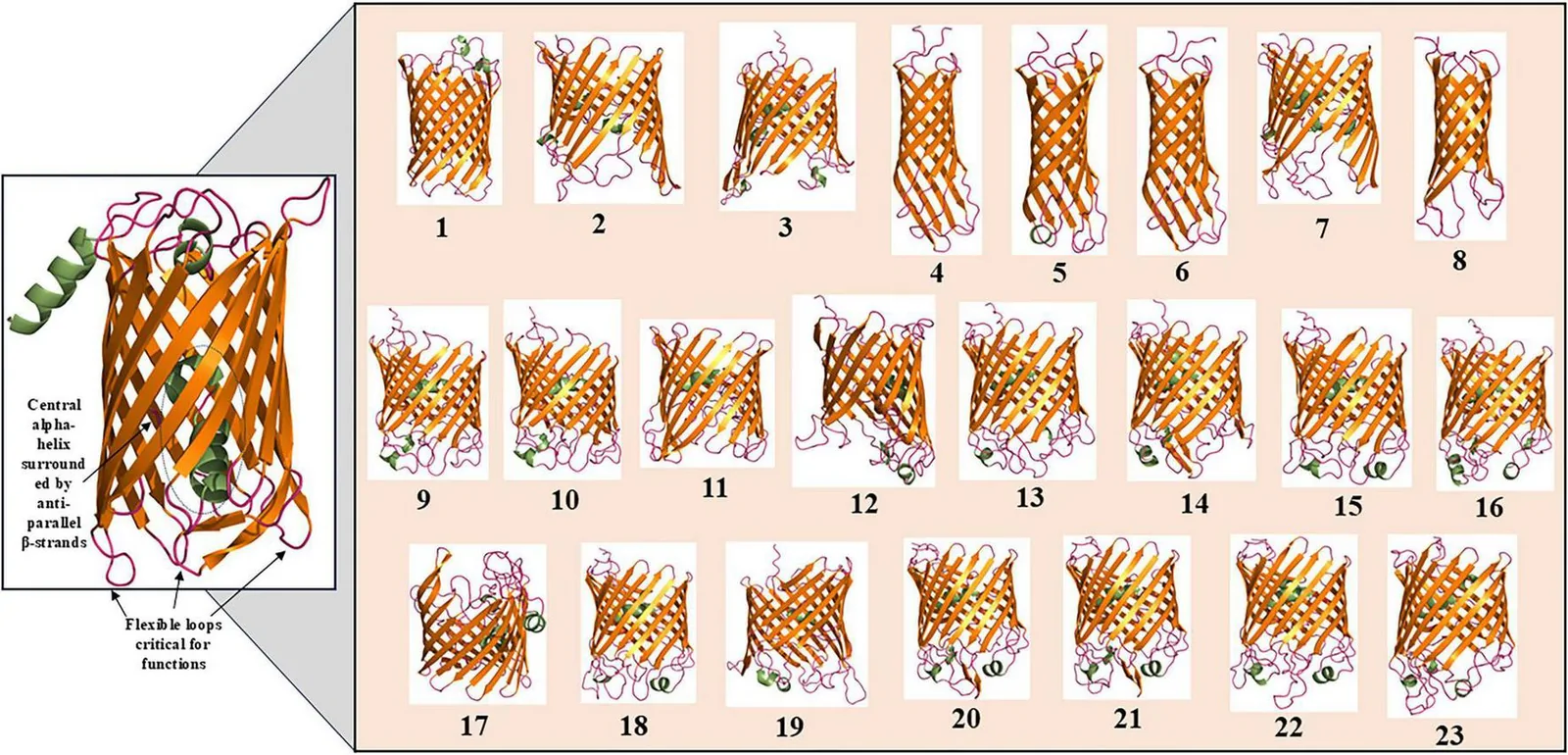
Comparative structural features of representative porins from Gram-negative bacteria. Ribbon representations of 23 optimized porins illustrate the conserved β-barrel scaffold and extracellular loop topologies across diverse Gram-negative species. Despite variation in sequence identity, all porins preserve the characteristic 14-18-stranded β-barrel architecture, aromatic girdle anchoring residues, and solvent-exposed loops that modulate channel selectivity. The reference strain *Acinetobacter baumannii* ATCC 19606 Omp33-36 model (center) is shown superimposed to highlight its canonical architecture and relative pore dimensions compared with homologues such as OmpF, OprD, and CarO.

The structural organization makes especially important consideration of the effects of sequence variation in the loop regions. A substitution in or near a loop does not necessarily have to disrupt the overall β-barrel fold to affect porin function but it might alter local steric constraints, interactions between residues, the flexibility of a loop or the spatial arrangement of residues around the entrance of a channel. Mutations that conserve the general β-barrel architecture may, however, alter the conformational behavior or accessibility of the pore. This distinction is particularly relevant to the Omp33-36 variants studied in the present study, where the identified substitutions lead to relatively localized sequence changes but may affect the behavior of loop-associated regions. Thus, Figure 1 provides the structural framework for subsequent modeling and molecular dynamics analyses of the clinical Omp33-36 variants.

#### 3.2.1 Effects of the Phe125Asn and Glu243Ser mutations

Using structural modeling and comparison of the study strain A_146 with the reference strain *A. baumannii* ATCC 19606 (Figure 2 and Supplementary Table 3), two key amino acid mutations were identified within the loop regions of the outer membrane porins Omp33-36. The first substitution was detected at position 125, where phenylalanine (Phe) was replaced by asparagine (Asn). The second alteration occurred at position 243, where glutamic acid (Glu) was substituted with serine (Ser) (Figure 3 and Supplementary Table 3). Both the mutations were detected in the loop regions.

**FIGURE 2:**
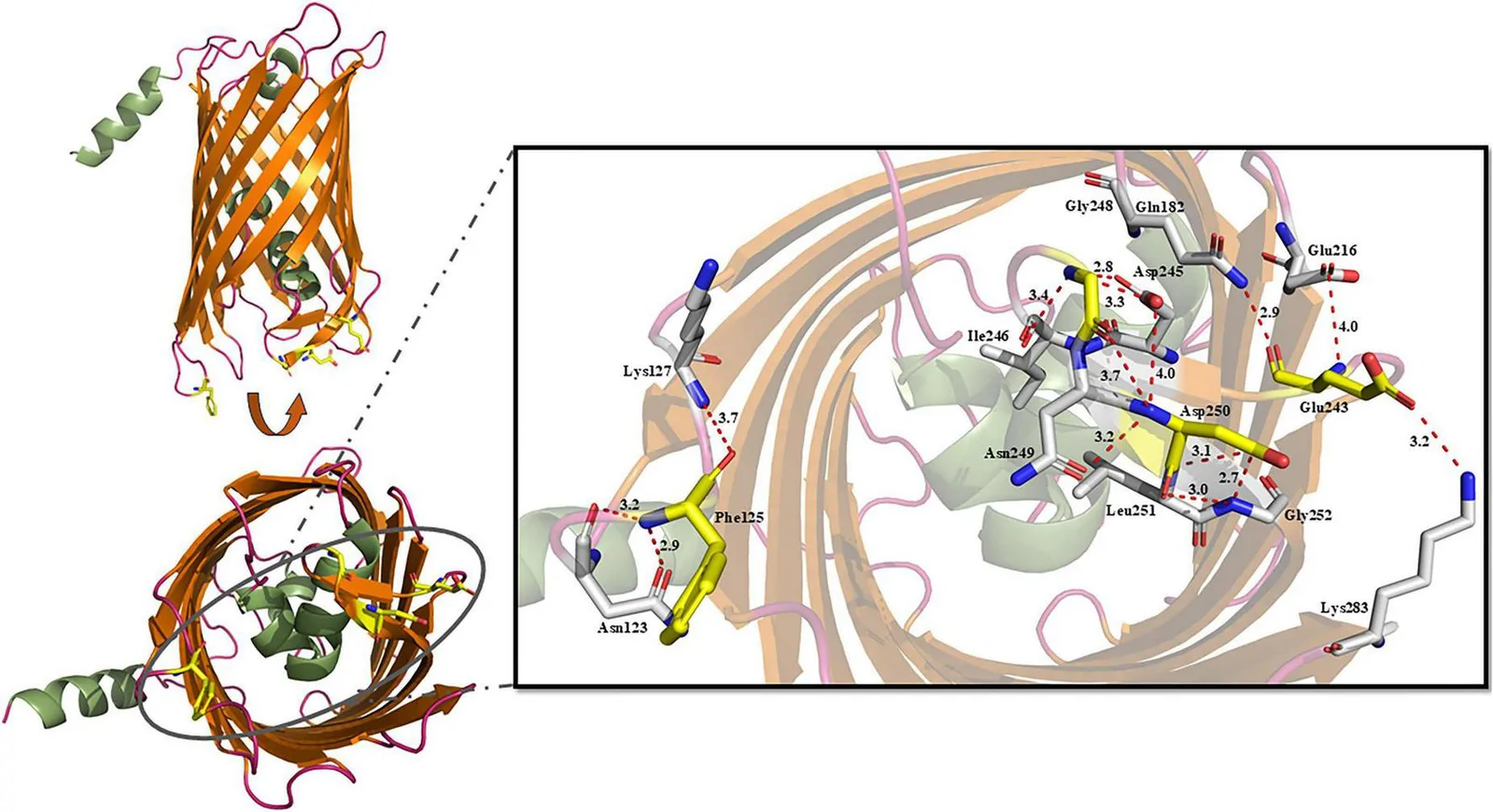
Structural mapping of loop-region mutations in the Omp33-36 of reference strain *Acinetobacter baumannii* ATCC 19606. Homology-modeled structure of wild-type Omp33-36 protein of the reference strain is shown in cartoon representation. The position of residues (mutated in strains A_146 and A_106) are represented as sticks (yellow), highlighting their proximity to the pore constriction zone and potential to alter loop-loop hydrogen-bonding networks.

**FIGURE 3:**
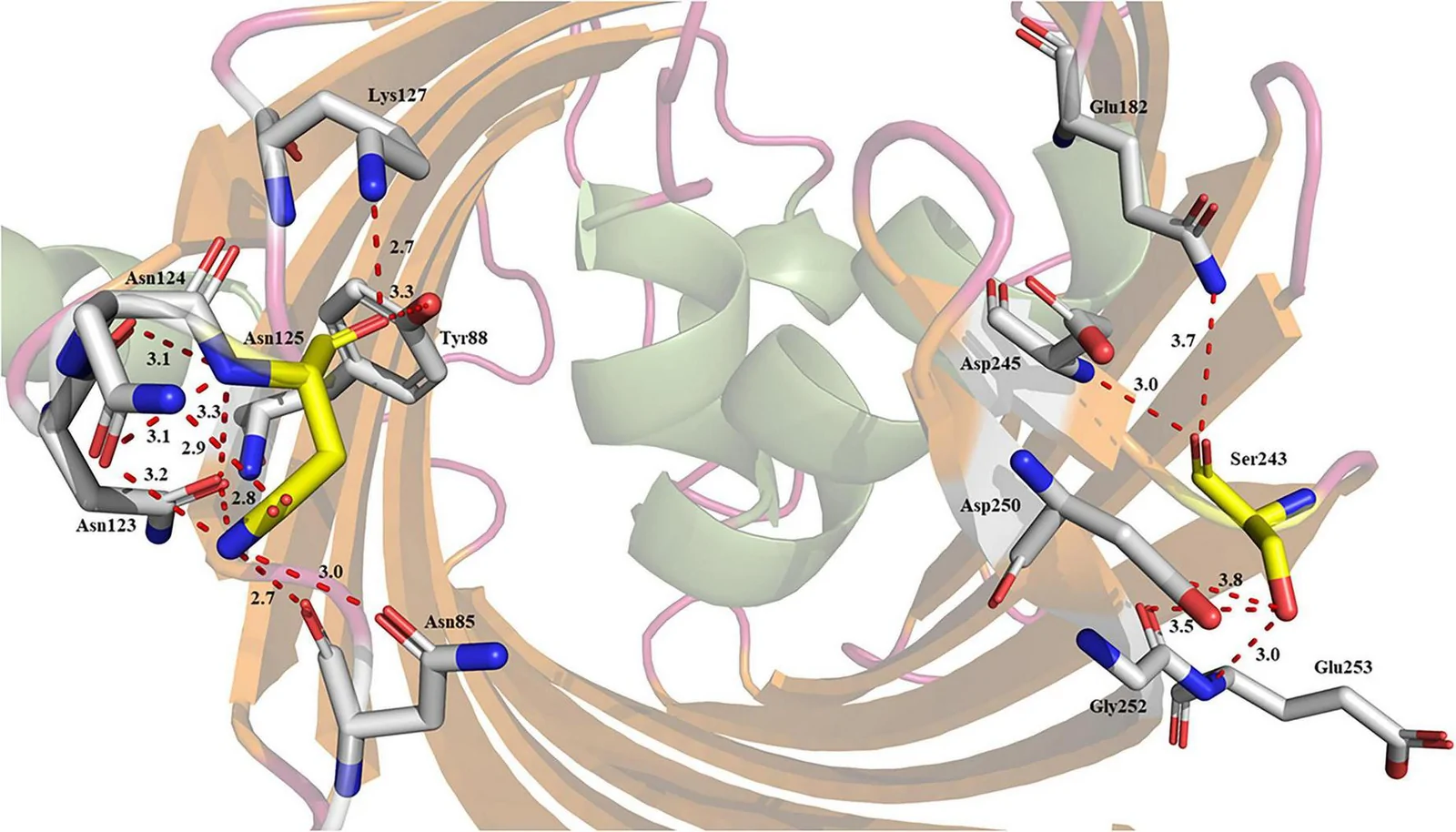
Structural consequences of Phe125Asn and Glu243Ser mutations in isolate A_146. Close-up view shows that Phe125 → Asn125 substitution introduces a polar side chain forming hydrogen bonds with Asp250, while Glu243 → Ser243 removes a negative charge and establishes short-range polar contacts with Asn245 and Thr247. Electrostatic surface comparison demonstrates narrowing of the solvent-accessible pore. Together, these substitutions generate cross-loop stabilization that stiffens the pore entrance and restricts solute permeation.

Phe125 was located in the loop connecting the cytosolic entry side (Asn123-Asp128) in the wild-type porin structure of the reference strain. The Phe125Asn substitution may alter the local interaction network through two sequential effects. In the wild-type protein, the bulky hydrophobic side chain of Phe125 was likely to impose a steric constraint that helps maintain separation between neighboring loop regions (Gly242-Gly252). Replacement of Phe125 by the smaller polar Asn residue removes this steric constraint, allowing greater local conformational rearrangement. This newly accessible configuration may then permit Asn125 to form a hydrogen-bonding interaction with Asp250. The resulting attractive polar interaction could favour closer association of the neighboring loops. Thus, rather than the mutation directly “pulling” the loops together, the proposed mechanism involves an initial loss of steric constraint, followed by a gain of a stabilizing polar interaction, which together may promote loop rapprochement and alter the geometry of the channel entry region.

Similarly, Glu243 in the wild-type porin was located in the loop that contains a β-hairpin spanning Gly242-Gly252. Glu243 was oriented so that its negatively charged side chain projected into the aqueous lumen. Consequently, the loop was stabilized by long-range electrostatic repulsion that maintained spacing between neighboring elements. Upon substituting Glu243 with Ser in the study strain A_146, the negative charge was lost, and a smaller hydroxyl-bearing side chain was introduced. Short-range polar interactions were subsequently formed between this hydroxyl group and neighboring residues such as Asn245 and Thr247, rendering the loop terminus less flexible and mechanically stiffer (Supplementary Table 3).

Together, these two mutations were found to mediate cross-loop hydrogen bonding and steric tightening, resulting in the stiffening and narrowing of one face of the porin barrel. The structural constriction thus produced likely impeded solute transport and reduced antibiotic permeability, potentially explaining the resistance observed in strain A_146.

#### 3.2.2 Effects of the Gly248Asp and Asp250Asn mutations

Two significant alterations (Gly248Asp and Asp250Asn) in the cytosolic entry-side loop (Gly242-Gly252) were identified when the structure of the study strain A_106 (Figure 4 and Supplementary Table 3) was compared with that of the reference strain porin (Figure 2 and Supplementary Table 3).

**FIGURE 4:**
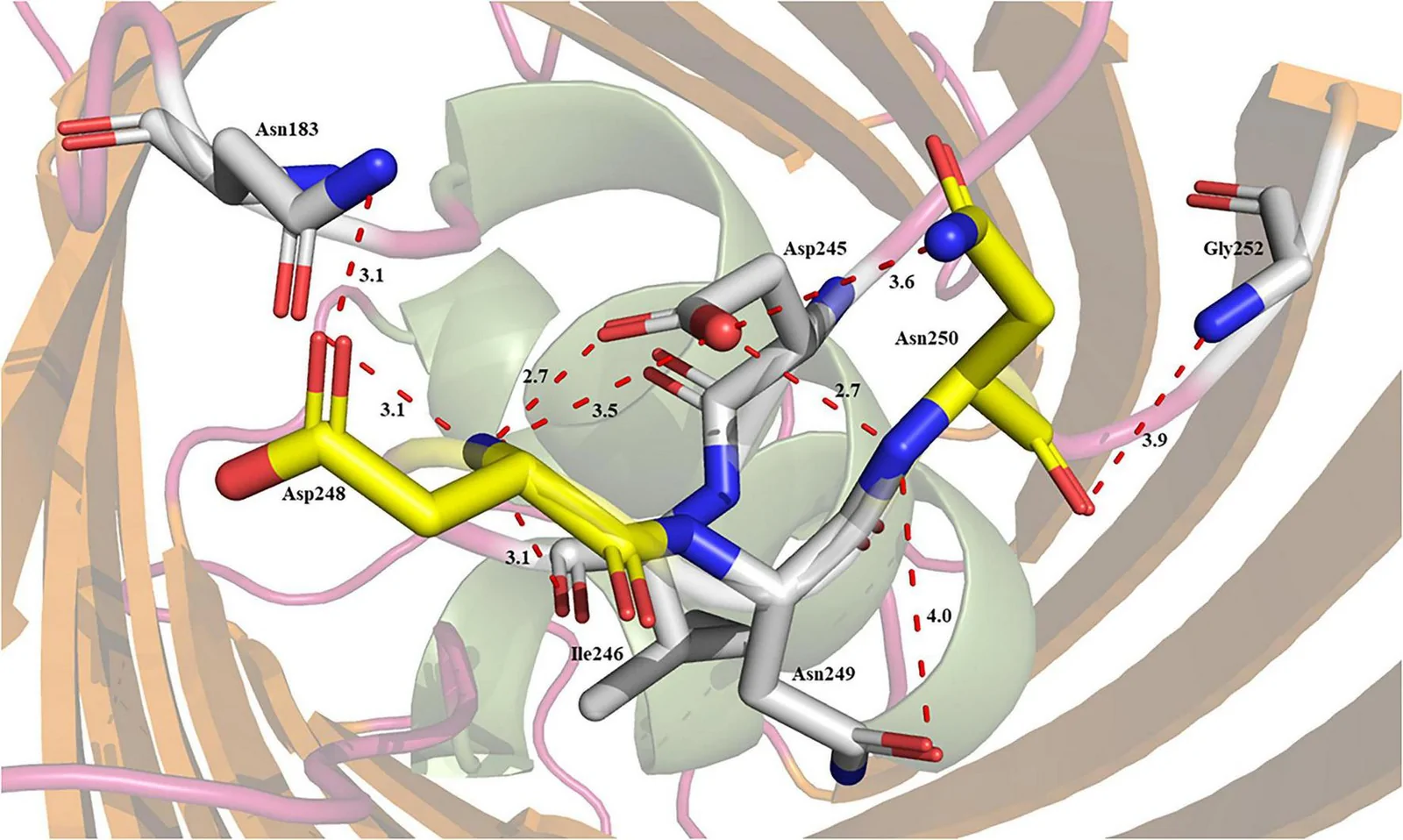
Structural effects of Gly248Asp and Asp250Asn mutations in isolate A_106. Close-up view shows pronounced loop restriction near the cytosolic entry side. Gly248 → Asp248 introduces a negatively charged side chain forming new hydrogen bonds with Gln215, Glu216, and Asn217. Asp250 → Asn250 neutralizes local charge and establishes cross-barrel hydrogen bonding with loops Gln158-Asp162 and Gln182-Gln185. The resulting inter-loop network reduces loop mobility, constricts the lumen, and diminishes antibiotic permeability.

In the wild-type Omp33-36 structure of the reference strain, this loop was relatively flexible and oriented in the opposite direction to loop Gln158-Asp162, remaining close to loop Gly214-Glu216. No stabilizing interactions were present at these positions. The glycine at position 248 imparted flexibility to the loop due to its minimal steric bulk, while the negatively charged Asp250 remained solvent-exposed, maintaining the porin barrel’s open lumen.

In the mutant Omp33-36 structure detected in A_106, the Gly248Asp substitution introduced a larger, negatively charged aspartic acid side chain. Structural modeling indicated the formation of novel hydrogen bonds and electrostatic interactions among residues Gln215, Glu216, and Asn217, adjacent to the loop comprising Gly214-Glu216. These interactions effectively anchored loop Gly242-Gly252 to its neighboring loop, restricting its conformational mobility.

The Asp250Asn substitution in A_106, replaced the negatively charged aspartate with a neutral but polar asparagine residue, facilitating hydrogen bond formation with residues in the opposite loops (Gln158-Asp162, Gln182-Gln185) and stabilizing the loop termini through polar interactions. As a result, the entrance-side loop (Gly242-Gly252) became linked to its opposing loop partners (Gln158-Asp162, Gln182-Gln185), rendering the cytosolic entry region more rigid.

The cumulative effect of these mutations created a network of inter-loop interactions: stabilization between Gly242-Gly252 and Gly214-Glu216 via Asp248, and cross-barrel stabilization among Gly242-Gly252, Gln158-Asp162 and Gln182-Gln185 via Asn250. This dual anchoring reduced loop flexibility, stiffened the cytosolic entry-side region of the β-barrel, and altered the structure in a manner that constricted the pore, decreased channel flexibility, and partially occluded one side of the lumen, thereby hindering antibiotic passage. These results corresponded with the observed phenotype of reduced permeability and resistance in strain A_106.

#### 3.2.3 Comparative analysis of Omp33-36 amino-acid variations in *A. baumannii* clonal lineages and different *Acinetobacter* spp

A comparative analysis of Omp33-36 amino-acid sequences detected in the study isolates A_146 (ST1406) and A_106 (ST1488) with those present in globally disseminated epidemic *A. baumannii* Clonal lineages and other *Acinetobacter* spp was performed (Supplementary Figure 3). ConSurf analysis showed that Omp33-36 contains a mixture of highly conserved and variable residues, with the four positions of interest (125, 243, 248, and 250) mapping to distinct regions of the porin structure. Notably, these mutation sites are predominantly located within or adjacent to surface-exposed loop regions and display variable-to-intermediate levels of evolutionary conservation. This suggests that these loop regions possess greater sequence plasticity than the highly conserved residues forming the β-barrel core. Such variability may permit amino-acid substitutions while potentially altering local loop conformation and pore-associated dynamics. In addition, the Omp33-36 sequences among the 25 *A. baumannii* strains belong to International Clone 2 (IC2/ST2) examined, were conserved at positions 125, 243, 248 and 250. However, the F125N and E243S substitutions found in the Omp33-36 sequence of A_146 were also present in an IC8/ST10 sequence, while D250N identified in A_106 was observed in the IC1/ST1 sequences. At position 248, the IC1/ST1 sequences contained G248A, whereas isolate A_106 contained G248D. Similarly, at position 125, the IC1/ST1 sequences contained F125V, whereas isolate A_146 contained F125N.

### 3.3 Analysis of membrane simulation experiment

Recognizing that static models might not fully capture the temporal evolution of these interactions, to produce further clarity, molecular dynamics (MD) simulations were conducted. The simulations confirmed the stability of the predicted hydrogen-bonding network and demonstrated restricted loop mobility over time. These findings corroborated that the amino acid mutations Gly248Asp and Asp250Asn in isolate A_106 and Phe125Asn and Glu243Ser in isolate A_146 jointly modified the structural mechanics of Omp33-36. These mutation-associated changes in Omp33-36 structure and dynamics may influence its permeability function and facilitate the antibiotic resistance phenotype of *A. baumannii*.

We employed all-atom MD simulations of full-length porins integrated into lipid bilayers, which faithfully reproduced physiologically relevant membrane compositions, to investigate the behavior of Omp33-36 porins. To capture the electrostatic and hydration effects critical to porin function, explicit water molecules and counterions were added to each system. It took considerable time to create and balance these protein-lipid-solvent complexes; however, this framework enabled examination of conformational dynamics at the atomic level.

Simulations were performed using the wild-type protein of the reference strain and two study strains: A_146 (with mutations Phe125Asn and Glu243Ser) and A_106 (with mutations Gly248Asp and Asp250Asn). Comparative trajectory analyses were conducted with an emphasis on metrics such as root mean square deviation (RMSD) for structural stability, root mean square fluctuation (RMSF) for residue-level mobility, and radius of gyration (Rg) for the overall compactness of the porin-membrane complexes. Simulations employing established force fields were used to assess how the newly formed hydrogen-bonding and electrostatic networks influenced loop mobility over time, particularly around the channel entrance. To visualize these complex dynamics, the simulation trajectories were converted into high-resolution molecular animations: wild-type Omp33-36 of the reference strain (Supplemental Movies 1, 2A and 2B), A_146 (Phe125Asn and Glu243Ser; Supplemental Movies 3A and 3B), and A_106 (Gly248Asp and Asp250Asn; Supplemental Movies 4A and 4B). In each MP4 file, the porin was embedded in a lipid bilayer and surrounded by solvent molecules, illustrating real-time hydrogen bonding, side-chain rotameric transitions, and loop rearrangements. These visualizations provided compelling evidence that specific side-chain substitutions altered interaction networks, reduced channel flexibility, and influenced porin function, thereby elucidating the molecular basis of resistance development in *A. baumannii*.

#### 3.3.1 Root mean square deviation (RMSD)

Root mean square deviation was conducted to assess global conformational fluctuations, reflecting changes in the global structural state of the protein during the trajectory. The RMSD trajectories for the Omp33-36 of the reference strain (black), study strain A_146 (red), and A_106 (blue) are shown in Figure 5. All three proteins were found to have attained equilibrium and structural stabilization during the simulation.

**FIGURE 5:**
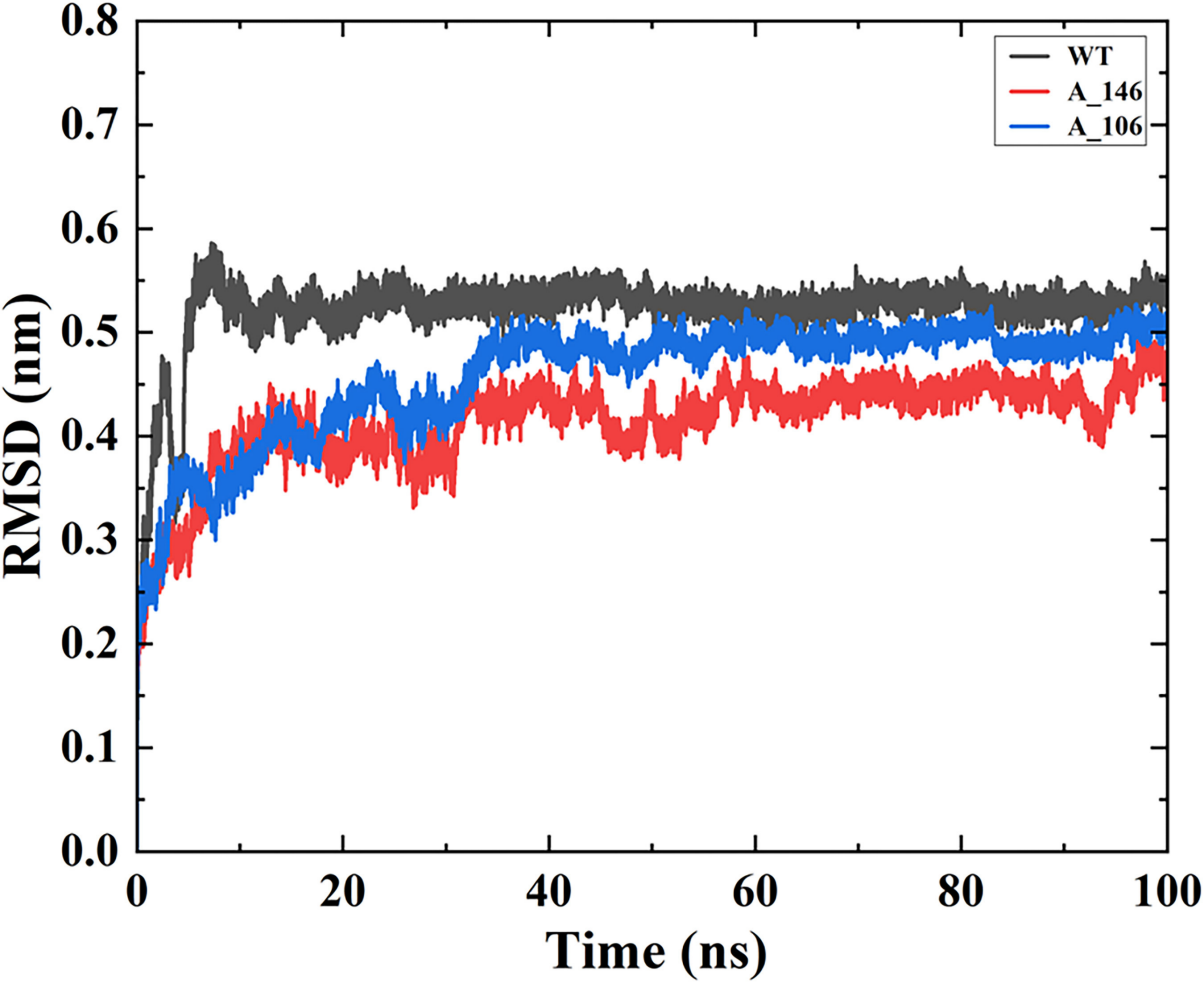
RMSD profile of global conformational stability of Omp33-36 and its variants A_146 and A_106. Root mean square deviation (RMSD) profiles show differences in the overall conformational fluctuations of wild-type *A. baumannii* ATCC 19606 (black) and mutant Omp33-36 proteins (A_146 as red, and A_106 as blue) during the 100-ns simulation.

The reference strain exhibited greater RMSD amplitudes than the study strains (Supplementary Table 4), indicating greater conformational fluctuation/deviation. This observation was consistent with the absence of additional stabilizing interactions in the reference strain, where unrestricted loop dynamics maintained a more adaptable pore conformation. In contrast, isolates A_146 and A_106 displayed reduced RMSD fluctuations (Supplementary Tables 5 and 6), corresponding to the formation of new hydrogen bonds and electrostatic interactions that limited loop mobility. The introduction of these non-covalent contacts increased the rigidity of the protein scaffold, thereby reducing global fluctuations and restricting pore mobility, which likely hindered translocation of antibiotics. The lowest RMSD values were recorded for the mutant proteins, suggesting that among the two study strains, isolate A_146 was more structurally stable than A_106.

#### 3.3.2 Root mean square fluctuation (RMSF) and B-factor

Root mean square fluctuation was used specifically to describe local residue mobility, with particular emphasis on the loop regions showing increased fluctuations in the mutant proteins. Figure 6A presents the RMSF plots for the reference strain (black), study strains A_146 (red), and A_106 (blue). Interestingly, although the reference strain exhibited higher global fluctuation (as shown by RMSD), certain regions, particularly residues 160–180, displayed lower RMSF values than the mutants which was associated with a lateral clearance event identified during the trajectory, in which the loop adopted a displaced configuration that increased the available space at the channel entrance. Trajectory analysis resolved this apparent paradox. In the reference strain, the loop encompassing residues 160-180 initially moved laterally away from the pore axis and then stabilized in a fixed position. This “loop clearance” event widened the channel lumen, facilitating antibiotic diffusion into the cytosol. Consequently, the immobilization of this loop reduced local residue fluctuations, producing lower RMSF values in this region (Supplementary Table 4).

**FIGURE 6:**
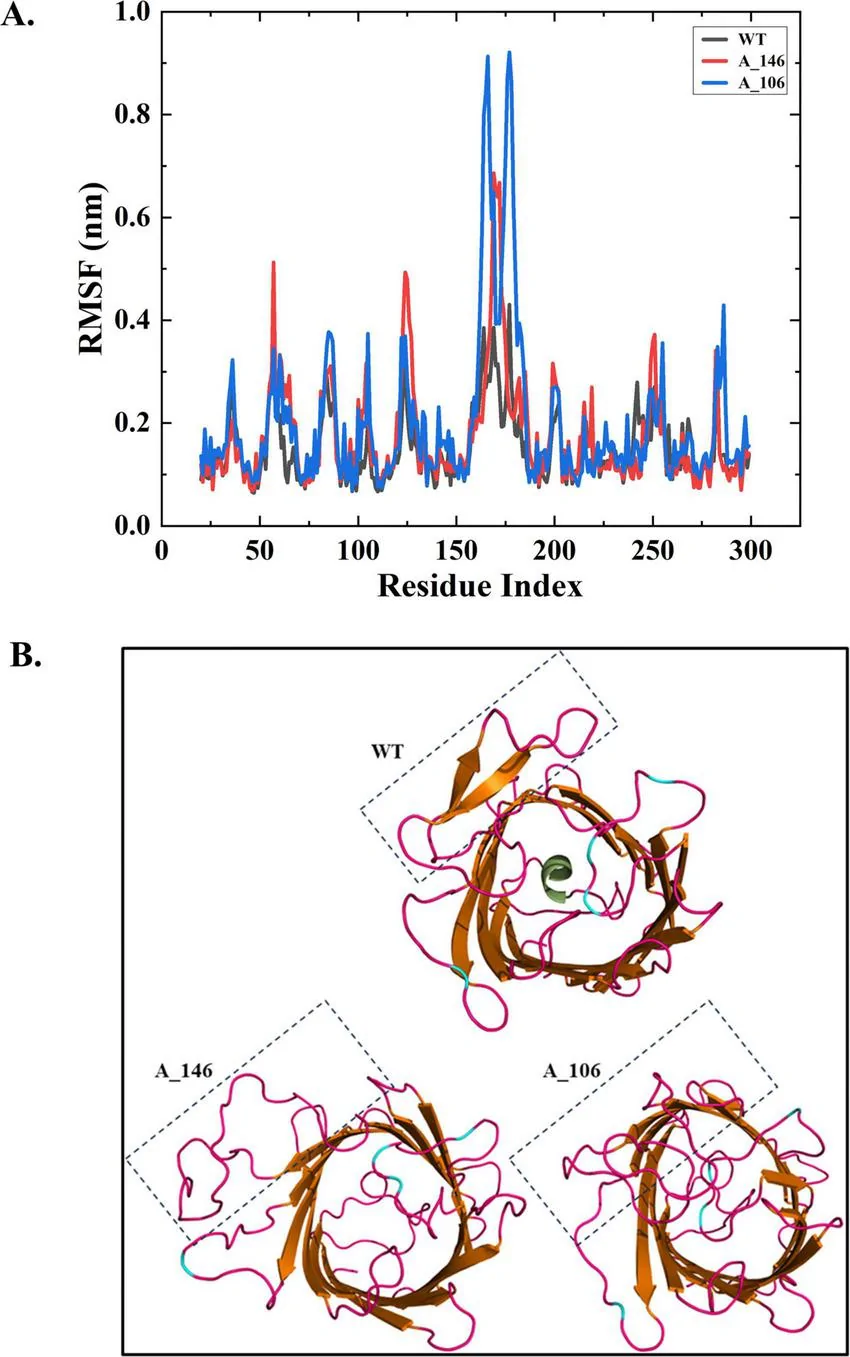
Residue-level dynamics of wild-type and mutant Omp33-36. **(A)** Root-mean-square fluctuation (RMSF) profiles of wild-type Omp33-36 from the reference strain *A. baumannii* ATCC 19606 (WT, black), A_146 carrying Phe125Asn and Glu243Ser substitutions (red), and A_106 carrying Gly248Asp and Asp250Asn substitutions (blue) during the 100-ns molecular dynamics simulations. The profiles show mutation-associated differences in local residue mobility, particularly around the loop regions involved in pore entry. **(B)** Representative snapshots of wild-type Omp33-36 from *A. baumannii* ATCC 19606 (WT) and the mutant proteins A_146 (Phe125Asn and Glu243Ser) and A_106 (Gly248Asp and Asp250Asn). Dashed boxes highlight the loop-associated regions surrounding the channel entrance. Wild-type loops remain retracted, maintaining an open lumen, whereas mutants show inward loop movement restricting the passageway.

In contrast, the modified loops in A_146 and A_106 did not disengage from the pore but remained dynamically active, undergoing frequent conformational changes near the channel entrance. The novel interactions, such as Asp248-Gln215/Glu216/Asn217 in A_106 and the Asn125-Asp250 bridging in A_146, acted as tethers, drawing the loops inward toward the pore lumen and periodically obstructing the channel. As a result, the mutants displayed higher RMSF values, reflecting higher-frequency local oscillations across the pore axis rather than sustained openness. Similarly, the mutant loop fluctuations are described as potentially influencing local pore accessibility rather than being presented as a direct measurement of pore occlusion or permeability. Representative snapshots illustrating this behavior are shown in Figure 6B. The wild-type Omp33-36 loop of the reference strain remained retracted, maintaining an open pore, whereas the loops in the study strains A_146 and A_106 progressively intruded into the pore cavity, dynamically restricting solute flow.

The mutant’s altered loop conformation does not seem to correspond to a single instantaneous displacement, but rather to an altered conformational state sampled along the MD trajectory (Supplementary Tables 5, 6). After mutation the local packing environment changes, releasing constraints that would otherwise keep the loop in its starting position, allowing the loop to move away from the channel entrance and to adopt a more ‘cleared’ conformation. In this state the loop forms a re-organized network of local contacts with neighbouring residues which may be involved in stabilizing the displaced orientation. The changed position may be further stabilized by the surrounding lipid environment, through additional steric and hydrophobic constraints at the membrane interface. In particular, the cleared configuration was seen several times along the trajectory and was not only an endpoint snapshot, indicating that the mutation promotes different conformational sampling of this region (Figure 7). However, since the occupancy and energetic contribution of single hydrogen bonds, residue-residue contacts or lipid interactions are not quantitatively determined in the present analysis, these interactions are to be considered as possible contributors and not as definitive stabilizing determinants. Therefore, the observed rearrangement could change the access and the geometry of the entrance of the channel, giving a structural basis to the proposed effect of the mutation on Omp33-36 function.

**FIGURE 7:**
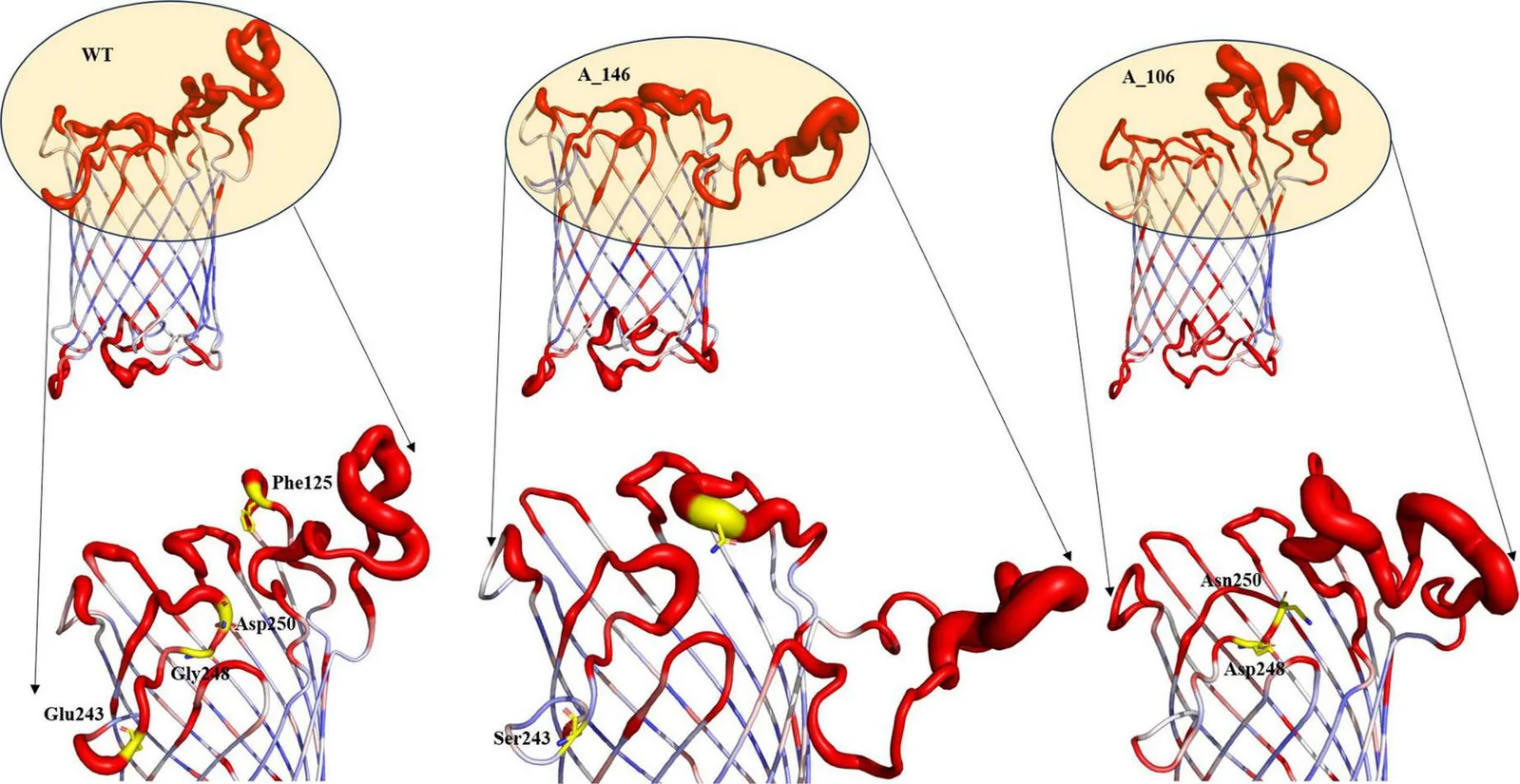
B-factor representation of wild-type (*A. baumannii* ATCC 19606) and mutant Omp33-36. B-factor-based putty representations of WT, A_146, and A_106 Omp33-36. The thickness of the protein backbone represents the relative B-factor, with thicker regions indicating greater positional fluctuation or flexibility. The mutated residues are highlighted in yellow. The representations emphasize the flexible extracellular loop regions and their altered structural environments in the mutant proteins.

#### 3.3.3 Radius of gyration (Rg)

The radius of gyration (Rg) was analysed to determine the compactness and folding stability of the structures. The Rg values for both the reference strain and study strains remained consistent at approximately 2.0 nm (±0.5 nm) throughout all simulation trajectories (Figure 8). These results confirmed that, despite localized loop rearrangements and transient pore occlusion events, the overall β-barrel framework of Omp33-36 remained structurally intact.

**FIGURE 8:**
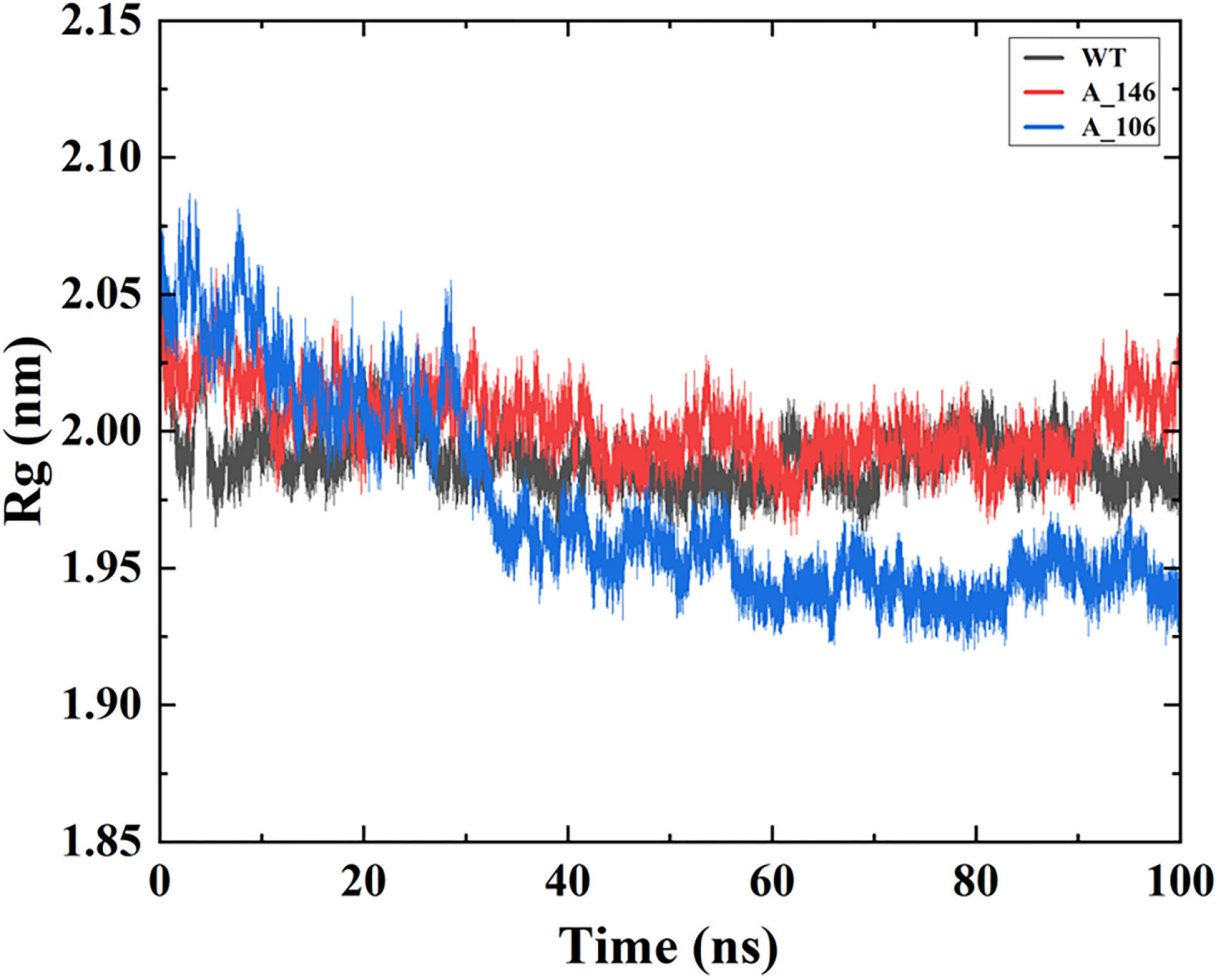
Radius of gyration of wild-type and mutant Omp33-36 during molecular dynamics simulations. Radius of gyration (Rg) profiles of wild-type Omp33-36 from *A. baumannii* ATCC 19606 (WT, black), A_146 carrying Phe125Asn and Glu243Ser substitutions (red), and A_106 carrying Gly248Asp and Asp250Asn substitutions (blue) over the 100-ns molecular dynamics simulations. The Rg values remained within a relatively narrow range throughout the simulations, indicating that the overall compactness of the Omp33-36 structures was maintained. Radius-of-gyration (Rg) plots for wild-type Omp33-36 and mutant porins indicate consistent global compactness (∼2.0 ± 0.5 nm) throughout simulations, confirming that mutations affect local loop rigidity without destabilizing the β-barrel scaffold.

The absence of significant fluctuations in Rg values indicated that neither the introduced mutations nor the resulting dynamic loop interactions compromised the porin’s global architecture. Therefore, the functional effects observed were attributed to localized loop rigidification and partial occlusion of the channel, rather than destabilization of the overall β-barrel scaffold.

### 3.4 Mechanistic interpretation of a proposed two-gate coupled-motion model for Omp33-36

In the proposed model, the term “two gates” refers to the two opposing access regions of the Omp33-36 transmembrane pathway and not to two individual gate-forming residues. One is the access region facing the extracellular environment where solutes approach the porin. The second is the opposite, periplasm-facing region, through which molecules enter into the periplasm. In the WT Omp33-36, the β-barrel serves as a relatively rigid scaffold, and the loop-associated regions are conformationally flexible. Their collective motions transiently open the pore laterally and expand it, thereby maintaining an accessible, hydrated permeation pathway. By “synchrony” we mean coupled conformational behavior of these spatially separated loop-associated regions, and not coupling via a single permanent hydrogen bond or hydrophobic hinge.

Indeed, clinical mutations appear to disrupt this dynamic balance by generating new local and cross-loop interactions. Phe125Asn in A_146 replaces a bulky hydrophobic side chain that contributes to steric separation with a polar residue that can form additional cross-loop contacts. Glu243Ser in A_146 removes a negative charge and favors short-range polar interactions. The Gly248Asp in A_106 introduces a larger charged side chain to tether the Gly242-Gly252 region to the neighboring Gly214-Glu216 region. Asp250Asn also forms polar interactions with the opposite Gln158-Asp162 and Gln182-Gln185 regions. These interactions act as molecular constraints restricting the mobility of the loop and promoting inward, pore restricting conformations. The mutations do not affect the β-barrel scaffold but instead alter the mechanical coupling between its dynamic loop regions, causing Omp33-36 to change from a flexible, fluctuating-in-concert state to a more rigid and less accessible conformational ensemble. This provides a structural rationale for the proposed reduction in antibiotic permeation, but direct validation of the two-gate coupling mechanism would require quantitative correlated-motion, pore-radius, hydration and permeability analyzes.

## 4 Discussion

Reduced outer-membrane permeability is a key factor contributing to antimicrobial resistance in Gram-negative bacteria. The decreased abundance of porins or alterations of the pore architecture can result in decreased access of antimicrobial molecules to their intracellular or periplasmic targets. This permeability barrier is part of a broader, inherently multifactorial resistance network in *Acinetobacter baumannii*. These include β-lactamases, drug-modifying enzymes, efflux systems and changes in outer membrane proteins. In this regard, Omp33-36 has raised interest due to its β-barrel architecture that is characterized by long and conformationally dynamic loop regions that may influence the accessibility of the transmembrane pathway. Here, we combine the characterization of clinical isolates with expression analysis of *omp33-36*, sequence analysis, comparative structural modeling, and molecular dynamics simulations of the full-length membrane-embedded protein to study the possible impact of naturally occurring Omp33-36 substitutions on the structural and dynamic behavior of this porin.

The two MDR bloodstream isolates examined have two different but potentially complementary alterations in Omp33-36. First, *omp33-36* was significantly downregulated compared to that in *A. baumannii* ATCC 19606, about 0.3-fold in A_146 and 0.2-fold in A_106. Second, sequencing showed Phe125Asn/Glu243Ser in A_146 and Gly248Asp/Asp250Asn in A_106. These results are consistent with the more general notion that porin-mediated resistance can occur either by quantitative changes in the number of channels due to the reduced expression (Clark, 1996; del Mar Tomás et al., 2005; Liu et al., 2012; Kyriakidis et al., 2021) or by qualitative changes in the properties of the remaining expressed channels due to the mutations (Catel-Ferreira et al., 2011; Gomez-Simmonds et al., 2018; Zhu et al., 2019; Prajapati et al., 2021). A decrease in expression alone should lead to a lower number of Omp33-36 molecules in the outer membrane and therefore could play a significant role in changes in permeability. The structural implications of the substitutions are to be interpreted in the light of this important expression level background, not as a separate explanation of the resistance phenotype.

Structural modeling suggests that A_146 and A_106 substitutions do not destabilize the basic β-barrel architecture, but remodel local interaction networks around loop-associated regions of the channel. This distinction matters because one can alter porin function without necessarily disrupting the barrel. Localized changes in steric packing, electrostatics, hydrogen bonding and conformational sampling around an access region may be sufficient to alter channel behavior.

In A_146, the Phe125Asn substitution changes a bulky hydrophobic aromatic residue to a smaller polar residue. The structural model indicates a sequential, not just attractive, mechanism. The aromatic side chain of Phe125 is freed from a local steric constraint that separates neighboring loop regions. This conformational freedom allows for alterations in the local environment and enables Asn125 to form additional polar interactions, including a proposed interaction with Asp250. Thus, the substitution can be thought of as a steric-to-polar switch: removal of the steric bulk allows the first loop to approach, which is followed by a reorganization of the hydrogen-bonding network that may favor the new configuration. The Glu243Ser substitution induces a reciprocal perturbation in the Gly242-Gly252 region. Replacing the negative glutamate with a smaller serine, which has a hydroxyl group, removes negative charge in the vicinity and allows polar interactions at a shorter distance with neighbouring residues. Thus, the modeling suggests that Phe125Asn and Glu243Ser act through different physico-chemical mechanisms. The former involves loss of steric bulk at one position and the latter redistribution of charge/polar interactions at the other; both, however, converge on the same structural region by reorganizing inter-loop contacts. The net result is not a global unfolding but a redistribution of local constraints in the channel-access region.

A_106 provides a more obvious example of mutation-induced cross-loop rewiring. Gly248Asp substitutes the conformationally permissive glycine with a larger negatively charged side chain and is predicted to mediate further interactions with residues around the neighboring Gly214-Glu216 region. Asp250Asn replaces a negatively charged residue with a neutral polar side chain capable of forming a different hydrogen-bonding network between opposing loop regions. The combination of these substitutions leads to better cohesiveness of the local network between the Gly242-Gly252 region and the surrounding structural elements. Moreover, further analysis of the interaction reveals a significant reorganization of the residue contacts around positions 248 and 250 compared to wild-type Omp33-36.

So, the key mechanistic story that both variants are telling us is not just “mutations make the protein rigid. Substitutions come at the expense of conformational freedom. They provide new possibilities of interactions in some loop regions, but the scaffold of the β-barrel is relatively stable. So relative loop motions might be limited, though individual residues or regions of the loop might fluctuate strongly on short timescales.

The MD simulations provide a crucial dynamic perspective to the static structural models. RMSD, RMSF and radius of gyration are probing different aspects of molecular behavior and should not be thought of as interchangeable. RMSD mainly reports changes in the overall conformational state against a reference structure. RMSF reflects local residue-level flexibility, while Rg reports global protein compactness and its changes over time. Thus, a protein can have a globally tight and intact β-barrel with large mutation-dependent changes in mobility of particular loops. The distinction is that it avoids the apparent paradox in the trajectories. For some of the mutant loop regions that show higher RMSF, it does not necessarily mean a more open or globally more flexible channel. Instead, trajectories show that the mutants’ sample different local conformational states around the pore-access region. The corresponding loop elements in the reference protein are displaced from the pore axis and in a relatively retracted conformation. In the variants, however, the regions associated with the loop still show higher-frequency local fluctuations around different interaction networks and may sample configurations closer to the channel pathway. We conclude that high local RMSF should be interpreted as oscillatory sampling in a geometrically constrained region, not as increased permeability. The manuscript trajectory analysis is consistent with this interpretation, and clearly distinguishes these local fluctuations from a direct measurement of pore occlusion or permeability. Globally, Rg was within a relatively narrow window of ∼2.0 ± 0.5 nm over the course of the simulations, supporting the overall compactness of Omp33-36 with local rearrangements. These observations are consistent with a model of local dynamic rewiring within a globally conserved β-barrel, rather than a mutation-induced destabilization of the entire porin.

This also limits the conclusions that can be drawn from the simulations. RMSD, RMSF and Rg describe the molecular conformation and dynamics and are not permeability measures. The present results show mutation-associated differences in the structural behavior of Omp33-36 but do not directly provide evidence of a reduced passage of antibiotics. Establishing such a relationship will require quantitative pore-radius profiling and solvent-accessible surface analysis, channel hydration and water occupancy, correlated-motion analysis, and finally molecular permeation or flux calculations and experimental transport measurements.

The structural and dynamic features can be incorporated in a working model of two-gate coupled-motion of Omp33-36. Here “gate” is not referring to two independent molecular valves. It concerns instead two spatially separated access regions on opposite sides of the transmembrane pathway, one extracellular-facing region by which a solute would approach the porin and one periplasm-facing region by which a permeating molecule would emerge. In each region, there are several loop and turn elements whose conformational behavior is supported by the relatively rigid β-barrel scaffold. In the wild-type model, these spatially separated dynamic elements are conformationally flexible enough to participate in concerted rearrangements, without permanently constraining the putative permeation pathway. This is called coupled motion, or dynamic synchrony. Importantly, synchrony does not imply a permanent mechanical coupling or the simultaneous opening and closing of two physical gates. Rather it is a working hypothesis in which conformational changes at one access region are integrated into the larger dynamic network of the porin.

These variants seem to influence this dynamic equilibrium. Phe125Asn/Glu243Ser/Gly248Asp/Asp250Asn alters the conformational states accessed by loop-associated regions by changing the local steric, electrostatic and hydrogen-bonding environments. Representative trajectories and structural snapshots show that the β-barrel remains largely intact, while mutation-associated changes are localized near the channel entrance. We suggest that the substitutions do not simply “close” Omp33-36, but rather remodel its conformational landscape and may bias the channel toward conformations with less favorable access along the transmembrane pathway.

This interpretation also allows a more sophisticated mechanistic framework than a static open versus closed pore model. Omp33-36 may be a case of a dynamically accessible pathway, where the permeability is not only a function of the average geometry of the pore but also dependent on the frequency, duration and coordination of the accessible conformational states. Thus, mutations that modulate the coupling between the loops could change the probability of sampling permeation-competent conformations, without creating a physical barrier.

The two-gate model is still a hypothesis based on the present structural and MD data. Correlated gating or translocation of antibiotics is not explicitly shown in the simulations. Quantitative dynamic cross-correlation or mutual information analyzes are necessary to determine if the motions at the two proposed access regions are statistically coupled.

One major strength of the present study is that the structural findings can be interpreted in the light of the actual genetic and phenotypic context of the clinical isolates. Both A_146 and A_106 were resistant to several antimicrobials and were MDR. Genotypic screening revealed the presence of *bla*_PER_ and *aac(6′)-Ib-cr* in both study isolates as previously reported in *A. baumannii* (Chatterjee et al., 2016; Roy et al., 2021). Thus, substitutions in Omp33-36 cannot be directly related to the antimicrobial resistance phenotype. The most plausible interpretation is a two-tiered contribution of Omp33-36 to a multifactorial resistance phenotype. Quantitatively, downregulation of *omp33-36* may translate into a decrease in the number of available channels. The substitutions in the residual porin population can affect the local loop interaction network and the conformational sampling at the qualitative level. These mechanisms could be cooperative, leading to a decrease in the number of channels and a modification of the dynamic properties of the remaining channels. However, since expression and sequence changes occur together in the clinical isolates, we are unable to disentangle the contribution of each separately with the experimental setup used here.

This distinction becomes especially relevant when considering the relationship between the MD results and susceptibility to antimicrobials. The simulations do not demonstrate that the substitutions induce the observed MIC phenotype, but they provide a plausible structural mechanism by which the substitutions may affect channel access. Therefore, Omp33-36 should be considered as a possible contributing determinant and not an independent demonstrated MDR driver in A_146 and A_106. This is also in line with the overall conclusion of the manuscript, which states that reduced porin expression and mutation-associated changes may act together to affect permeability against a background of additional resistance determinants.

To place the Omp33-36 variants identified in the sporadic ST1406 and ST1488 isolates into a broader epidemiological context, we compared their deduced amino-acid sequences with representative sequences from globally disseminated *A. baumannii* clonal lineages and different *Acinetobacter* spp. Interestingly, the 25 representative IC2/ST2 Omp33-36 sequences examined were highly conserved at the four positions (125, 243, 248 and 250) altered in our isolates, indicating that the substitutions observed in A_146 and A_106 are not characteristic of the IC2/ST2 background. In contrast, variation at these positions was evident in other epidemic lineages. In particular, an IC8/ST10 sequence contained the same F125N and E243S substitutions identified together in A_146, whereas the IC1/ST1 sequences contained D250N, corresponding to one of the substitutions in A_106, although residue 248 (G248A in IC1/ST1 versus G248D in A_106) and 125 (F125V in IC1/ST1 versus F125N in A_146) differed. Thus, these substitutions are not necessarily unique to the sporadic ST1406 and ST1488 isolates but can occur in genetically distinct *A. baumannii* backgrounds. Moreover, comparison with other *Acinetobacter* spp., showed that the four amino-acid positions (125, 243, 248 and 250) altered in our isolates belong to the variable region. This observation suggests that positions 125, 243, 248 and 250 constitute variable regions of Omp33-36 whose functional consequences may depend on both the specific amino-acid replacement and its surrounding structural context. Accordingly, our molecular-dynamics results should be interpreted as defining the structural and dynamic consequences of the particular substitution combinations present in A_146 and A_106, rather than as demonstrating lineage-specific resistance determinants.

Several factors limit the present conclusions. First, MD simulations describe structural dynamics and not molecular transport: Antibiotic permeation through Omp33-36 was neither directly simulated nor experimentally measured. Second, the sharp drop in *omp33-36* expression in both isolates does not permit to distinguish mutation-specific effects from a decrease in porin abundance. Third, there are other acquired resistance determinants present, so it is not possible to attribute the MDR phenotype specifically to Omp33-36. Fourth, the individual hydrogen bonds, residue-residue contacts and lipid interactions observed during the trajectories were not quantified in terms of occupancy or energetic decomposition and are therefore to be considered as candidate contributors to the changed conformational behavior, not as definitive molecular determinants (Hazra and Dubey, 2022).

Future validation should therefore be both at the experimental level and computational level. Isogenic strains expressing wild-type or single Omp33-36 variants from a regulated genetic background would enable the effects of sequence variation to be separated from differences in porin abundance. Functional validation can be carried out through complementation experiments, antimicrobial susceptibility measurements and direct outer-membrane permeability assays. Computationally, time-resolved pore-radius profiling, solvent accessibility, channel hydration and water occupancy, dynamic cross-correlation analysis and molecular permeation/free-energy calculations could tell if the mutation-associated conformational states found here translate into quantitatively reduced channel accessibility (Dhankhar et al., 2016).

To summarize, the current study supports a model whereby Omp33-36 substitutions remodel the porin without destabilizing it. Gly248Asp/Asp250Asn and Phe125Asn/Glu243Ser modify local networks of steric and polar interactions and influence the sampling of conformations associated with the loop region, while preserving the global beta-barrel fold (Bharatiy et al., 2016). These results, together with the strong down-regulation of the omp33-36 expression, suggest that Omp33-36 could be involved in the permeability related resistance profile of the tested *A. baumannii* strains. The coupled-motion model of two gates presented here provides a testable framework to link localized loop dynamics to channel accessibility, differentiating this structural hypothesis from direct evidence of antibiotic transport. Future studies of permeability and molecular flux in isogenic strains are needed to determine how much this dynamic remodeling contributes to antimicrobial resistance in *A. baumannii*.

## 5 Conclusion

Antimicrobial resistance is rarely the result of a single molecular event. Instead, it is the outcome of the bacteria’s capacity to continually reconfigure the relation between the cell and the antimicrobial molecules attempting to enter. Our work provides a convincing structural example of how apparently small changes at the outer membrane can contribute to this larger adaptive landscape in multidrug-resistant *A. baumannii*. We resort to a combination of clinical isolate characterization, *omp33-36* expression analysis, mutation mapping at the sequence level, comparative structural modeling and full-length membrane-embedded molecular dynamics simulations to bridge the gap between a simple observation of porin alteration and a mechanistic framework of how such changes might affect channel behavior. The clinical isolates complemented each other in two ways: First, expression of *omp33-36* was significantly reduced in both the isolates compared to the reference strain, presumably resulting in fewer available porin channels. Secondly, the other Omp33-36 molecules had different loop associated substitutions and structural analyzes suggest that such substitutions do not destabilize the basic β-barrel scaffold. Rather, they remodel the local molecular environment by changing steric constraints, charge distribution, hydrogen bonding and cross loop interactions. The molecular dynamics simulations contribute an important dimension to the structural picture. The mutations do not simply make Omp33-36 “more rigid” or “more flexible” globally but appear to redistribute its conformational freedom. The β-barrel is globally compact with Rg values within a relatively narrow range, although some loop regions show altered local fluctuations and conformational sampling. This differentiation correlates the local RMSF behavior with structural evidence for altered loop interactions and provides an important mechanistic idea: porin modification associated with resistance could entail not channel destruction, but the rewriting of its dynamic landscape. On the basis of these observations, we propose a two-gate coupled-motion model in which the access regions facing the extracellular and periplasmic sides of Omp33-36 function as spatially separated, but dynamically coordinated components of the same trans-membrane pathway. It will require testing by direct pore-geometry, hydration, correlated-motion, molecular-flux and experimental permeability measurements. The biological story is therefore more complex – and perhaps more interesting – than one mutation leading to resistance.

## Data Availability

Sequencing data has been deposited in NCBI GenBank under the accession numbers PZ511637 and PZ511638.

## Footnotes

1
https://pubmlst.org/organisms/acinetobacter-baumannii

## Supplementary material

Supplementary Figure 1 Ramachandran plot analysis of the modeled Omp33-36 structures. Ramachandran plots showing the backbone dihedral angle distributions of the wild-type Omp33-36 from *A. baumannii* ATCC 19606 (WT), and the Omp33-36 variants from clinical isolates A_146 and A_106. The plots show the distribution of residues in the most favoured, additionally allowed, generously allowed, and disallowed regions. More than 90% of the residues in all three models were located in the most favoured regions, supporting the overall stereochemical quality of the modeled structures and their suitability for subsequent molecular dynamics simulations.

Supplementary Figure 2 Multiple sequence alignment of Omp33-36 and representative porin-like proteins from Gram-negative bacteria. Multiple sequence alignment of Omp33-36 (WP_000733005.1) from *Acinetobacter baumannii* ATCC 19606 with representative outer-membrane porins and porin-like proteins from different Gram-negative bacteria. Conserved and variable amino acid positions across the aligned sequences are shown, with gaps represented by dots. The alignment illustrates the conserved sequence features shared among these β-barrel outer-membrane proteins while also highlighting sequence variation within regions corresponding to extracellular loops and other structurally relevant regions of Omp33-36. The protein identifiers and accession numbers are indicated alongside the sequences.

Supplementary Figure 3 Evolutionary conservation and comparative sequence analysis of Omp33-36 from *Acinetobacter baumannii*. (A) ConSurf analysis showing the evolutionary conservation of Omp33-36 residues, with the positions of interest (125, 243, 248, and 250) highlighted in the sequence/structural context among the *Acinetobacter* species. (B) Multiple-sequence alignment of Omp33-36 from the study isolates and representative epidemic *A. baumannii* lineages (IC1/ST1, IC2/ST2, and IC8/ST10), generated using MultAlin and visualized with ESPript. The positions corresponding to residues 125, 243, 248, and 250 are highlighted to show their conservation and lineage-associated sequence variations.

Supplementary Movie 1 Molecular dynamics simulation (100 ns) of wild-type Omp33-36 of the reference strain of *A. baumannii* in a DPPC bilayer showing dynamic loop motions that maintain an open, hydrated pore.

Supplementary Movie 2A Side view of wild-type Omp33-36 36 of the reference strain of *A. baumannii* illustrating coordinated oscillation of surface loops controlling pore opening and hydration.

Supplementary Movie 2B Top view of wild-type Omp33-36 36 of the reference strain of *A. baumannii* showing stable β-barrel architecture with limited motion of periplasmic loops.

Supplementary Movie 3A Side view of the study isolate A_146 (Phe125Asn, Glu243Ser) revealing restricted loop motion and pore narrowing due to new hydrogen-bond interactions.

Supplementary Movie 3B Top view of the study isolate A_146 showing reduced flexibility and a constricted channel entrance compared with the wild type porin.

Supplementary Movie 4A Side view of the study isolate A_106 (Gly248Asp, Asp250Asn) demonstrating loop stiffening and lumen constriction through cross-loop anchoring.

Supplementary Movie 4B Top view of the study isolate A_106 highlighting reduced loop mobility and partial occlusion of the pore lumen.
