# Supplementary material for: Loop region mutations in Omp33-36: unveiling reduced antimicrobial permeability and resistance in Acinetobacter baumannii through structural modeling and membrane-protein simulations
Source: Front Microbiol. 2026 Oct 20;17:1954565. doi: 10.3389/fmicb.2026.1954565 (PMC13727541; doi:10.3389/fmicb.2026.1954565)
Supplement: Supplementary file 1 [file Table_1.docx]

**Loop Region Mutations in Omp33-36: Unveiling Reduced Antimicrobial Permeability and Resistance in *Acinetobacter baumannii* through Structural Modelling and Membrane-Protein Simulations**

Subhasree Roy ^a,$^, Khalid Ansari^b,c,$^, Mousumi Hazra^d^, Vivek Junghare ^b,e^, Tanusree Das^a^, Arup Samanta^c^, Sulagna Basu^a*^ and Saugata Hazra^b,f,*^

$ Both authors contributed equally.

^a^Division of Bacteriology, ICMR National Institute for Research in Bacterial Infections (ICMR-NIRBI), Kolkata-700010, India

^b^Department of Bioscience and Bioengineering, Indian Institute of Technology Roorkee (IITR), Roorkee, Uttarakhand 247667, India

^c^Department of Physics, Indian Institute of Technology Roorkee (IITR), Roorkee, Uttarakhand 247667, India

^d^Department of Botany and Microbiology, Gurukula Kangri (Deemed to be University), Haridwar-249404, Uttarakhand, India

^e^Centre of Excellence for Chemical Mechanisms of Life, Uppsala University, Sweden

^f^Centre for Nanotechnology, Indian Institute of Technology Roorkee (IITR), Roorkee, Uttarakhand 247667, India

***Address for correspondence**:

Dr. Saugata Hazra, Associate Professor, Department of Bioscience and Bioengineering, Indian Institute of Technology-Roorkee, Roorkee, Uttarakhand, India; Telephone no.:
+918171064462; Email: saugata.iitk@gmail.com and saugata.hazra@bt.iitr.ac.in.
ORCID ID: 0000-0002-3074-1534

Dr. Sulagna Basu, Scientist G, Division of Bacteriology, ICMR-National Institute for Research in Bacterial Infections, Kolkata, India; Email: basus.niced@gov.in. ORCID ID: 0000-0002-7811-7140

**Table S1: Details of Simulation Systems.**

| **Simulation candidate** | **Number of Lipids** | **Number of Waters** | **Number of Ions** | **Total number of Atoms** |
| --- | --- | --- | --- | --- |
| Wild type | 89 | 6087 | 11 Na^+^ | 25500 |
| Isolate A_146 | 87 | 6190 | 10 Na^+^ | 25700 |
| Isolate A_106 | 88 | 6113 | 11 Na^+^ | 25534 |

**Table S2: Different porins from Gram-negative bacteria were compared sequentially and structurally against the Omp33-36 protein sequence of a wild type strain *A. baumannii* ATCC 19606 (WP_000733005.1)**

| **Sl.No.** | **Seq_name** | **Organism** | **Length** | **Template** |
| --- | --- | --- | --- | --- |
| 1 | 1_EcOmpG_P76045 | Escherichia coli (strain K12) | 301 | 7Q5C |
| 2 | 2_NmPorB1_P30690 | Neisseria meningitidis serogroup B (strain ATCC BAA-335) | 331 | 3WI5 |
| 3 | 3_VcOmpU_A5F934 | Vibrio cholerae serotype O1 (strain ATCC 39541) | 341 | 6EHB |
| 4 | 4_KpOmpA_P24017 | Klebsiella pneumoniae | 344 | 5NHX/2K0L |
| 5 | 5_EcOmpA_P0A910 | Escherichia coli (strain K12) | 346 | 9FZC/2MQE |
| 6 | 6_SfOmpA_A0A2S4N3N0 | Shigella flexneri | 348 | 3NB3 |
| 7 | 7_NgPorB1_P18195 | Neisseria gonorrhoeae | 348 | AlphaFold |
| 8 | 8_PaOprF_P13794 | Pseudomonas aeruginosa (strain ATCC 15692 / DSM 22644) | 350 | 5U1H/4RLC |
| 9 | 9_StPhoE_P30705 | Salmonella typhimurium (strain LT2 / SGSC1412 / ATCC 700720) | 350 | AlphaFold |
| 10 | 10_EcPhoE_P02932 | Escherichia coli (strain K12) | 351 | 1PHO |
| 11 | 11_DaOmp32_P24305 | Delftia acidovorans (Pseudomonas acidovorans) (Comamonas acidovorans) | 351 | 2FGR |
| 12 | 12_KpPhoE_P30704 | Klebsiella pneumoniae | 351 | AlphaFold |
| 13 | 13_EcOmpF_P02931 | Escherichia coli (strain K12) | 362 | 8C7L |
| 14 | 14_StOmpF_P37432 | Salmonella typhimurium (strain LT2 / SGSC1412 / ATCC 700720) | 363 | 3NSG |
| 15 | 15_KpOmpC_Q48473 | Klebsiella pneumoniae | 363 | 1OSM |
| 16 | 16_EcNmpC_P21420 | Escherichia coli (strain K12) | 365 | AlphaFold |
| 17 | 17_HaHopP_A9ZM27 | Halomonas sp | 366 | AlphaFold |
| 18 | 18_EcOmpC_P06996 | Escherichia coli (strain K12) | 367 | 8I8X |
| 19 | 19_BaPUsgL_Q8K9I8 | Buchnera aphidicola subsp. Schizaphis graminum (strain Sg) | 369 | AlphaFold |
| 20 | 20_SfOmpC_A0A2S4MYF8 | Shigella flexneri | 373 | 3NB3 |
| 21 | 21_EcOmpC_Q8CVW1 | Escherichia coli O6:H1 (strain CFT073 / ATCC 700928 / UPEC) | 375 | 2XE1 |
| 22 | 22_EcOmpN_P77747 | Escherichia coli (strain K12) | 377 | AlphaFold |
| 23 | 23_StOmpC_P0A264 | Salmonella typhi | 378 | 3UPG |

**Table S3: The comparison between isolate A_146 and A_106 in Omp33-36 and wild type indicating mutated residue and its interacting residues.**

| **Wild Type** | | | | |  | **Isolate A_146** | | | | |
| --- | --- | --- | --- | --- | --- | --- | --- | --- | --- | --- |
| **Residue** | **Interacting Atom** | **Residue** | **Interacting Atom** | **Distance**  **(Å)** |  | **Residue** | **Interacting Atom** | **Residue** | **Interacting Atom** | **Distance**  **(Å)** |
| Phe125 | O | Lys127 | N | 3.7 |  | Asn125 | O | Lys127 | NZ | 2.8 |
| Phe125 | N | Asn123 | OD1 | 2.9 |  | Asn125 | O | Tyr88 | OH | 3.3 |
| Phe125 | N | Asn123 | O | 3.2 |  | Asn125 | N | Asn123 | O | 3.1 |
|  |  |  |  |  |  | Asn125 | N | Asn123 | OD1 | 3.3 |
|  |  |  |  |  |  | Asn125 | N | Asn124 | OD1 | 3.1 |
|  |  |  |  |  |  | Asn125 | OD1 | Asn124 | ND2 | 2.9 |
|  |  |  |  |  |  | Asn125 | ND2 | Asn124 | OD1 | 3.2 |
|  |  |  |  |  |  | Asn125 | ND2 | Asn123 | OD1 | 2.8 |
|  |  |  |  |  |  | Asn125 | ND2 | Asn85 | OD1 | 3.0 |
|  |  |  |  |  |  | Asn125 | ND2 | Asn85 | O | 2.7 |
|  |  |  |  |  |  |  |  |  |  |  |
| Glu243 | O | Glu182 | NE2 | 2.9 |  | Ser243 | O | Gln182 | NE2 | 3.7 |
| Glu243 | N | Glu216 | OE1 | 4.0 |  | Ser243 | O | Asp245 | N | 3.0 |
| Glu243 | OE1 | Lys283 | NZ | 3.2 |  | Ser243 | OG | Glu253 | N | 2.0 |
|  |  |  |  |  |  | Ser243 | OG | Gly252 | O | 3.5 |
|  |  |  |  |  |  | Ser243 | OG | Asp250 | OD1 | 3.8 |
|  | | | | | | | | | | |
| **Wild Type** | | | | |  | **Isolate A_106** | | | | |
| **Residue** | **Interacting Atom** | **Residue** | **Interacting Atom** | **Distance**  **(Å)** |  | **Residue** | **Interacting Atom** | **Residue** | **Interacting Atom** | **Distance**  **(Å)** |
| Gly248 | N | Ile246 | O | 3.4 |  | Asp248 | N | Ile246 | O | 3.1 |
| Gly248 | N | Asp245 | OD1 | 2.8 |  | Asp248 | N | Asp245 | OD1 | 2.7 |
| Gly248 | N | Asp245 | OD2 | 3.3 |  | Asp248 | N | Asp245 | OD2 | 3.5 |
| Gly248 | O | Asp250 | N | 3.7 |  | Asp248 | N | Asn183 | OD1 | 3.1 |
|  |  |  |  |  |  | Asp248 | N | Asn183 | ND2 | 3.1 |
|  |  |  |  |  |  |  |  |  |  |  |
| Asp250 | N | Asp245 | OD2 | 4.0 |  | Asn250 | O | Gly252 | N | 3.8 |
| Asp250 | N | Asn249 | OD1 | 3.2 |  | Asn250 | N | Asn249 | OD1 | 4.0 |
| Asp250 | O | Gly252 | N | 3.7 |  | Asn250 | N | Asp245 | OD2 | 2.7 |
| Asp250 | OD1 | Gly252 | N | 2.7 |  | Asn250 | ND2 | Asp245 | OD2 | 3.6 |
| Asp250 | OD1 | Leu251 | N | 3.1 |  |  |  |  |  |  |

**Table S4: Omp 33-36 trajectories snapshots of Wild Type at different frames**

|  | **Protein without Lipid Bilayers** |  | **Protein without Lipid Bilayers** |
| --- | --- | --- | --- |
| **At frame 0** |  | **At frame 1000** |  |
|  | **A** |  | **B** |
| **At frame 2000** |  | **At frame 3000** |  |
|  | **C** |  | **D** |
| **At frame 4000** |  | **At frame 5000** |  |
|  | **E** |  | **F** |
| **At frame 20,000** |  | **At frame 50,000** |  |
|  | **G** |  | **H** |
|  |  |  |  |
|  | **I; Top view (with lipid bilayers)** |  | **J; Bottom view (with lipid bilayers)** |
|  |  |  |  |
|  | **K; Side view (with lipid bilayers)** |  | **L; Side view (with lipid bilayers and waters)** |

**Table S5: Omp33-36 trajectories snapshots of isolate A_146 at different frames**

|  | **Protein without Lipid Bilayers** |  | **Protein without Lipid Bilayers** |
| --- | --- | --- | --- |
| **At frame 0** |  | **At frame 1000** |  |
|  | **A** |  | **B** |
| **At frame 2000** |  | **At frame 3000** |  |
|  | **C** |  | **D** |
| **At frame 4000** |  | **At frame 5000** |  |
|  | **E** |  | **F** |
| **At frame 25,000** |  | **At frame 50,000** |  |
|  | **G** |  | **H** |
| **At frame 50,000** |  | **At frame 50,000** |  |
|  | **I; Top view** |  | **J; Bottom view** |
| **At frame 50,000** |  | **At frame 50,000** |  |
|  | **K; Top view (with lipid bilayers)** |  | **L; Bottom view (with lipid bilayers)** |
| **At frame 50,000** |  | **At frame 50,000** |  |
|  | **M; Top view (with lipid bilayers and water)** |  | **N; Bottom view (with lipid bilayers and water)** |

**Table S6: Omp33-36 trajectories snapshots of isolate A_106 at different frames**

|  | **Protein without Lipid Bilayers** |  | **Protein without Lipid Bilayers** |
| --- | --- | --- | --- |
| **At frame 0** |  | **At frame 1000** |  |
|  | **A** |  | **B** |
| **At frame 2000** |  | **At frame 3000** |  |
|  | **C** |  | **D** |
| **At frame 4000** |  | **At frame 5000** |  |
|  | **E** |  | **F** |
| **At frame 20,000** |  | **At frame 50,000** |  |
|  | **G** |  | **H** |
| **At frame 50,000** |  | **At frame 50,000** |  |
|  | **I; Top view (with lipid bilayers)** |  | **J; Bottom view (with lipid bilayers)** |
| **At frame 50,000** |  | **At frame 50,000** |  |
|  | **K; Side view (with lipid bilayers)** |  | **L; Side view (with lipid bilayers and waters)** |
